# Tumor‐Mimic Artificial Cell Integrated With In Situ Synthetic Biology for Testing of Antitumor Drug Sensitivity

**DOI:** 10.1002/EXP.20240134

**Published:** 2026-05-28

**Authors:** Chaoyang Guan, Runchi Zhang, Zhihui Zhou, Pei Sun, Yichun Mao, Changqing Mao, Yonggeng Ma, Guifang Chen, Qiuhong Man, Chang Feng

**Affiliations:** ^1^ Center For Molecular Recognition and Biosensing Shanghai Engineering Research Center of Organ Repair Joint International Research Laboratory of Biomaterials and Biotechnology in Organ Repair (Ministry of Education), Shanghai Key Laboratory of Bio‐Energy Crops, School of Life Sciences, Shanghai University Shanghai China; ^2^ Department of Clinical Laboratory Medicine Shanghai Tenth People's Hospital, School of Medicine, Tongji University Shanghai China; ^3^ Department of Clinical Laboratory Medicine School of Medicine Shanghai Fourth People's Hospital Tongji University Shanghai China

**Keywords:** antitumor drugs, artificial cells, detection, efficacy, sensitivity

## Abstract

The novel models represented by organoids are becoming the key approach to solve the ethical and efficiency problems in drug development, but the effective and low‐cost models are still urgently needed. The breakthrough development of artificial cell (AC) technology and synthetic biology has made it possible. In this study, a novel AC for evaluating the efficacy of various antitumor drugs is fabricated by combining cell membrane bionic technology and in situ synthetic biology. After entering ACs, antitumor drugs targeting nucleic acid affect the gene transcription of an artificially designed ribozyme that can catalyze the cleavage of molecular beacons and generate fluorescence signals in situ, indicating the efficacy of antitumor drugs at the cellular level. Specifically, ACs constructed with cell membranes containing drug‐resistant proteins show significant drug inhibition, and the 3D coded ACs established based on this method are capable of classifying cell‐specific characteristics more accurately to provide support for targeted drug therapy. This platform for in situ pharmacodynamic analysis not only demonstrates the individualized penetration ability of tumor heterogeneous packaging membranes in response to tumor drugs but also fills the gap between non‐living and living experiments as a supplementary strategy.

## Graphical abstract

A biomimetic artificial cell model platform integrating in situ synthetic biology is developed, which can show antitumor drug response to simulated cells derived from cell membranes of different tumor cells. It has excellent performance in indicating the curative effect of drugs at the cellular level and provides a novel approach for enhancing the pertinence and effectiveness of clinical tumor treatment.

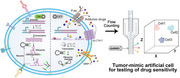

## 1 Introduction

Drug testing and evaluation are indispensable components of new drug development, following a progressive approach from biochemical analysis to cellular, animal, and human levels. At the biochemical level, widely used strategies include high‐performance liquid chromatography (HPLC) [1] and capillary electrophoresis (CE) [2]. At the cellular level and in vivo model levels, drug sensitivity test methods such as MTT colorimetric [3] and ATP bioluminescence technology (ATP‐TCA) [4] offer the advantages of rapidity and simplicity. Patient‐derived tumor xenograft (PDX) models closely mimic the real tumor environment in the human body and can accurately reflect the pharmacokinetic characteristics of the tested drugs [5]. Subsequently, the efficacy and safety of drugs can be effectively evaluated at the animal and human levels. The implantation of a drug depot into the patient's tumor enables the controlled release of various drugs or drug combinations in a spatially and temporally regulated manner [6]. Consequently, a rigorous testing and evaluation process has become an important basis for drug development. As technology advances, better protocols are breaking the traditional drug testing and evaluation process. For example, at the biochemical level, the development of artificial intelligence has brought new technical approaches to drug analysis [7, 8, 9]. By utilizing machine learning (ML) and deep learning (DL), intermolecular docking and potential drug screening are realized, significantly increasing the efficiency of new drug research and development and offering the potential for cost reduction and improved efficacy. At the animal and human level, the emergence of organoids has partially addressed ethical concerns related to animal and human experimentation. Organoids can simulate the tumor microenvironment in the human body and tumors’ heterogeneity over time, providing a more objective and accurate assessment of drug efficacy and safety [10, 11, 12]. However, the existing organoids exhibit high variability and lack fidelity and repeatability [13, 14]. Additionally, the cultivation of organoids is costly and time‐consuming, and their ability to simulate human organs is limited, especially in brain tissue [15, 16].

At the cellular level, the cells represent the smallest functional units with a comprehensive drug response mechanism. Considering the low cost and absence of ethical concerns associated with cell experiments, cells serve as ideal models for testing the sensitivity of antitumor drugs. Nonetheless, natural cells often lack readable signals and are subject to various interfering factors that can lead to plasmid loss or prolonged response signals [17]. Artificial cells (ACs) are structures designed and created using natural or synthetic materials to resemble biological cells [18]. The progress of synthetic biology has facilitated the development of ACs with specific functions for artificial regulation [19]. Simple artificial cells exhibit fundamental functions in living organisms, including metabolism [20], self‐replication [21], adhesion [22] and communication [23]. In practical applications, ACs demonstrate unique advantages in clinical settings, such as targeted transport function and drug delivery function by genome modification, protein engineering, and other technologies [24, 25]. In addition, ACs are currently employed to detect clinically relevant biomarkers as well as heavy metals and chemical contaminants, utilizing packaging transcription and translation systems, which holds significant promise for cancer treatment and environmental remediation [26, 27, 28]. However, due to the lack of self‐organization and self‐adaptability, it is still one of the challenges to design more stable and complex ACs, and the tools and technologies of synthetic biology are still incomplete, such as the unpredictability of the promoter‐reporter gene module. As a result, ACs have not been fully developed into innovative models for tumor treatment and evaluation based on cell levels.

The cell membrane constitutes a barrier for cells against the external environment and plays an important role in various physiological processes of cells, such as signal transduction, cell transport, cell survival, and differentiation [29]. As the special protective layer for cancer cells, cell membrane is the initial contact site for the drugs upon release [30, 31]. In the process of exerting effects on antitumor drugs, genes are regarded as the direct clinical targets clinically [32, 33], and the screening models and evaluations have been established accordingly [34]. In addition, the clinical trials of broad‐spectrum drugs that directly act on genes directly in various tumor indications have brought new treatment schemes for those malignant tumors that have no effective treatment drugs at present. Therefore, integrating the tumor cell membrane, which represents the initial response to tumor drugs, with artificially designed genes that serve as the direct targets of many tumor drugs, and simplifying them into an artificial cell system to generate output signals, undoubtedly holds significant value for the efficacy analysis of antitumor drugs. It is expected to construct a brand‐new artificial drug screening model. In this study, we constructed ACs using cell membrane biomimetic technology and in situ synthetic biology to evaluate the efficacy of different antitumor drugs. Using a tumor cell membrane encapsulating a transcription and ribozyme reporting system, nucleic acid‐targeted antitumor drugs enter artificial cells and affect the gene transcription of an artificially designed ribozyme that can catalyze the cleavage of molecular beacons and generate fluorescence signals, thus indicating the efficacy of antitumor drugs at different concentrations. This pharmacodynamic analysis platform based on artificial cells not only reveals the relationship between tumor heterogeneity and early adaptive drug resistance but also fills the gap between non‐living experiments and living experiments as a supplementary means, further promoting the specificity, pertinence, and effectiveness of clinical tumor treatment.

## 2 Results and Discussion

### 2.1 Principle of Tumor‐Mimic Artificial Cells for Antitumor Drug Testing

**FIGURE 1:**
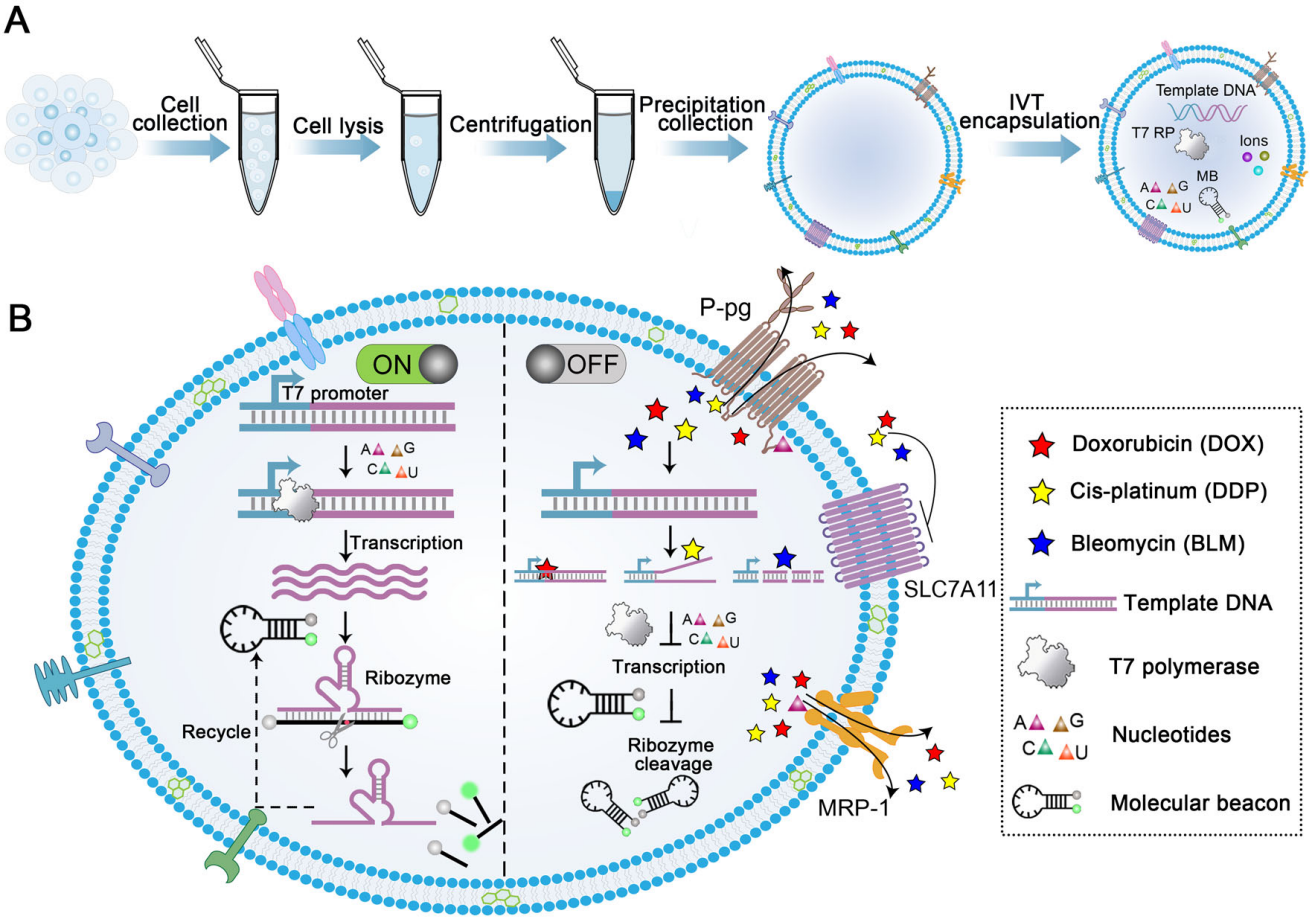
Schematic illustration of OPRS@CCMAC synthesis and its reflection on antitumor efficacy. (A) The construction process of CCMACs. (B) Schematic diagram of CCMACs for testing of antitumor drug sensitivity.

Cell membranes extracted from cancer cells have been endowed with the intrinsic characteristics of original cells and control their interactions with surrounding cells and tissues [35, 36]. The existence of multiple drug‐resistant transmembrane transporters on cancer cell membranes (CCM), such as p‐glycoprotein (P‐gp/ABCB1) [37, 38], multidrug resistance protein 1 (MRP‐1/ABCC1) [39, 40], and solute carrier family 7 member 11 (SLC7A11/xCT) [41, 42], is the key reason for the difference in sensitivity to anticancer drugs between primary and recurrent tumors. In this work, we have constructed two types of artificial cells simulating properties of natural cell surfaces, namely liposome‐coated ACs (LACs) and CCM‐coated ACs (CCMACs). Figure 1 shows the detailed principle of using CCMACs for antitumor drug sensitivity testing [43]. The drug sensitivity test model we proposed mainly consists of two independent processes: preparation of membrane materials and in vitro one‐pot reactions. As shown in Figure 1A, based on the previous work, we improved the membrane extraction procedure and obtained derived cell membranes with various transport membrane proteins from tumor cells [44]. In detail, we collected tumor cells in the logarithmic phase with a scraper, cracked them repeatedly with chemical reagents and the freeze‐thaw method, and isolated cell membrane fragments by high‐speed centrifugation. Subsequently, we resuspended these fragments in PBS solution and stored them at ‒80°C. Finally, we extracted an appropriate quantity of cell membrane fragments at a time for ultrasound to package the in vitro transcription (IVT) system and fabricate artificial cells.

Then, the IVT system was used to respond to different concentrations of doxorubicin (DOX), cisplatin (DDP), and bleomycin (BLM) (Figure 1B), which is composed of a double‐stranded DNA template, T7 RNA polymerase (T7 RP), nucleotide triphosphate (NTP), ions, and substrate probes (named MB). In the absence of antitumor drugs, the T7 promoter binds to the T7 RNA polymerase, which activates transcription in the downstream reporter region and produces hammerhead ribozyme (HHRz) [45]. HHRz is an ideal RNA trans‐cleavage tool that can specifically cleave fluorescent and quencher‐labeled MB to produce a green fluorescence signal output. However, in the presence of antitumor drugs, different concentrations of DOX, BLM, and DDP can enter into artificial cells through free diffusion [46, 47, 48]. Among them, DOX can be inserted into the DNA helix, and BLM can non‐specifically cut off the DNA template, while DDP can cross‐link the DNA template [49, 50], resulting in the failure of normal transcription of the DNA template, which produces an “OFF” fluorescence signal. Considering the multiple drug‐resistant transmembrane transporters on CCM can resist drug influx or efflux of internal drugs with the assistance of ATP, exerting the common mechanism of drug resistance, the damage of drugs to the DNA template was reduced, generating more fluorescence signals. The artificial cell model platform we constructed can quantitatively analyze the drug sensitivity of different multidrug‐resistant tumors, which provides a novel research approach for the communication between pharmacy and analysis.

### 2.2 Feasibility Validation of Testing Antitumor Drugs in Solution

**FIGURE 2:**
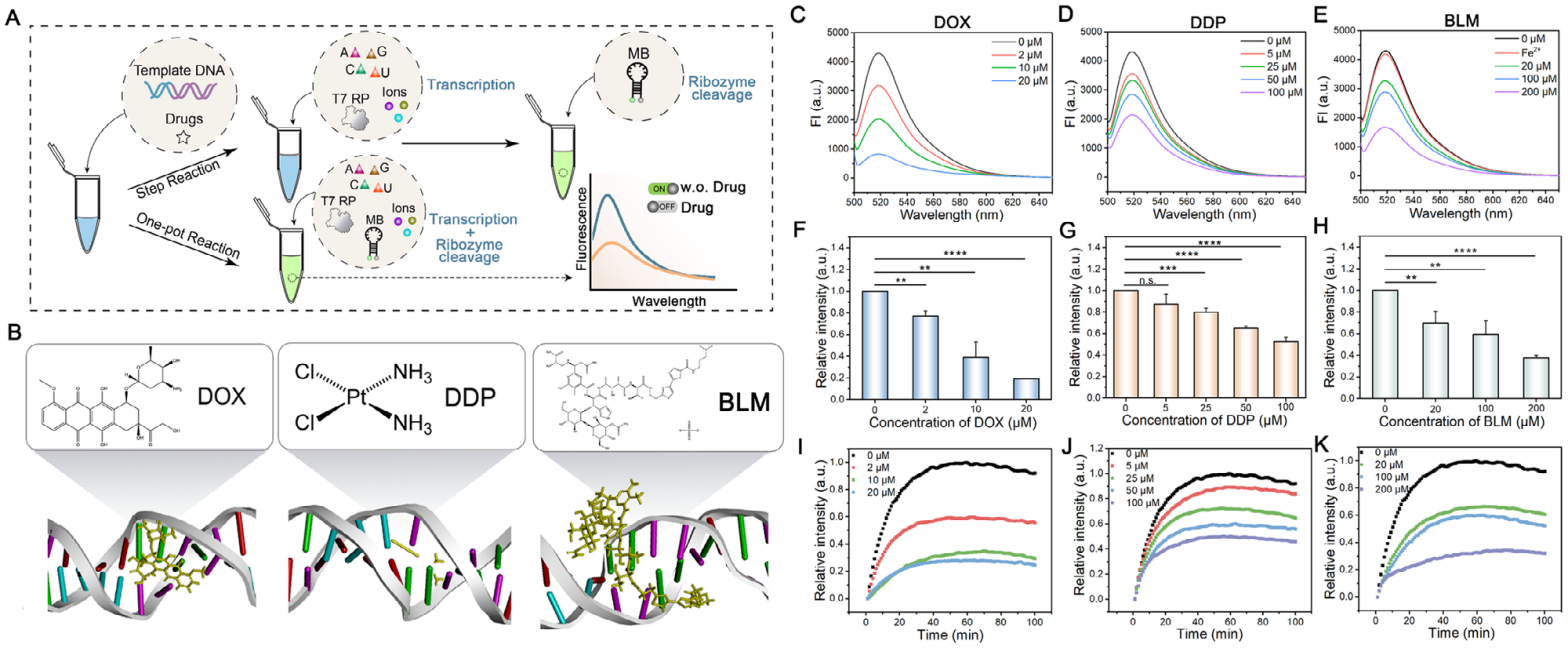
Characterization and sensitivity test of antitumor drugs in solution. A) Schematic illustration of drug sensitivity tests performed by step reaction and one‐pot reaction in solution. B) Molecular docking result of three drugs and double‐stranded DNA obtained by Discovery Studio 2018. C‐E) Fluorescence spectra of different concentrations of DOX, DDP, and BLM. F‐H) The difference of corresponding normalized fluorescence intensity at different concentrations of DOX, DDP, and BLM by step reaction in solution. I‐J) Fluorescence kinetics characterization of one‐pot reactions in solution at different concentrations of DOX, DDP, and BLM. In F‐H, all the points represent the means based on three independent replicates; bars represent the s.d. Statistical significance was determined by a two‐tailed student's t‐test; **p < 0.01, ***p < 0.001, and ****p < 0.0001; n.s., not significant (*p >* 0.05).

We first used the IVT system to test three antitumor drugs (DOX, DDP, and BLM) in solution. Figure 2A shows the steps involved in drug testing. In the step‐by‐step method, we first incubated double‐stranded DNA templates with different concentrations of drugs. Then after adding the IVT system, the undamaged DNA template was transcribed under the function of T7 RP to produce hammerhead ribozyme products. Subsequently, the substrate probe was added, and the ribozyme could specifically cleave MB to release green fluorescence. Figure 2B shows the molecular structures of three drugs (DOX, DDP, and BLM) and their simulated complexes interacting with template double‐stranded DNA. The results of molecular docking research display that the three drugs have good affinity and stability with the target DNA. The effect of the three drugs on DNA structure is evaluated by circular dichroism spectroscopy, and the results are shown in Figure S1. As it is observed, the intensities of both the negative and positive bands increase due to base destacking as DOX intercalation occurs. Whereas the intensity of both the bands undergoes obvious decreasing (shifting to zero levels) after interaction with DDP and BLM, suggesting that the binding of drugs to DNA induces conformational changes. The fluorescence spectrum showed that as the concentration of drugs in the solution increased, the released fluorescence signal reduced gradually, indicating that the drug can inhibit the generation of the fluorescence signal. Among them, the fluorescence signal of DOX decreased significantly at 20 µM. The signal of DDP and BLM decreased slowly at low concentrations and showed extremely significant fluorescence reduction at high concentrations (Figure 2C‒H). Previous studies have shown that the effect of BLM requires the involvement of an equal concentration of ferrous ions [51]. We also demonstrated that the interference background of ferrous ions on DNA can be ignored (Figure 2E). The above results are consistent with the verification of polyacrylamide gel electrophoresis (PAGE) (Figure S2), indicating that the IVT system we established can achieve the detection of antitumor drugs. In order to shorten the reaction time, by combining the in vitro transcription with the enzyme digestion system, we adopt a one‐pot reaction step to evaluate the efficacy for the detection of antitumor drugs in Figure 2A. We selected the same drug concentration as the step method and conducted real‐time fluorescence monitoring of the one‐step reaction process, as shown in Figure 2I‒K. The fluorescence kinetic curves of three different concentrations of drugs showed that the fluorescence signal of the reaction system gradually increased with the prolongation of time but reached equilibrium when the reaction was carried out for 60 min. Of note, the fluorescence signal ratio of the same drug concentration at 60 min has a good consistency with that of the step method, indicating that the one‐pot method can replace the step method, which is more time‐saving and convenient to operate, providing an available strategy for subsequent drug detection by artificial cells.

### 2.3 Construction and Characterization of LACs for Detecting Drug Sensitivity

Commercial liposomes composed of phospholipids are universally incubated with nucleic acids or proteins in the culture medium, which are used as transfection reagents to deliver nucleic acids or proteins to target cells [52]. Considering that the components in the medium may impact IVT and digestion systems, DEPC water was treated as buffer solution for the construction of LACs. In Figure S3, flow cytometry was used to explore the effect of different a buffers on liposome‐encapsulated DNA fluorescent probes. We distinguished the FAM‐labeled single‐stranded DNA probe (FAM‐DNA template) as the experimental group and the DNA template as the control group. Under the same test conditions, the liposome‐encapsulated DNA fluorescent probe based on DEPC water has more particles and a stronger fluorescence signal. Therefore, we use DEPC water as a preferred buffer for encapsulating IVT materials of the one‐pot method in situ (Figure S4A). Then characterization of LACs was performed using fluorescence microscopy, transmission electron microscope (TEM), and dynamic light scattering (DLS). By using lipophilic dye Dil to stain the OPRS@LACs, the outer membrane and interior of LACs show orange and green, respectively, in Figure S4B. The results indicated that the transcription‐enzyme digestion reaction is successfully carried out in LACs, and the released FAM green fluorescence is co‐localized with the orange fluorescence region of the liposome. In addition, the results of TEM in Figure S4C showed the regular spherical structure of LACs that is consistent with the dynamic light scattering (DLS, Figure S4F) method, and the particle size is mostly concentrated at 300 nm. In order to further demonstrate the ability of LACs to encapsulate DNA/RNA and active enzymes, we separately fabricated two liposomes containing FAM‐Template DNA and green fluorescent protein (GFP) in DEPC‐treated water with the same operation. DLS distribution showed that the particle size distribution of both liposomes was around 342 nm (Figure S4D) and 295 nm (Figure S4E), similar to OPRS@LACs, and the corresponding fluorescence microscopy images (Figure S5) of the two liposomes also visually confirmed the above results.

In order to obtain the optimal encapsulation efficiency, the conditions of the LACs preparation process were optimized, including liposome dosage, encapsulation time, and reaction time (Figure S6). Here, considering the variability of vesicle population [53, 54], we set up the same events in advance to control the variables to maintain other conditions consistent, and all in situ readouts of flow cytometry were calculated by mean fluorescence intensity (MFI). First, we measured the content of membrane materials that could completely encapsulate the in vitro transcription‐enzyme digestion system. Figure S6A and S6D show that MFI is stable after 1 µL, and excessive content will quench fluorescence. This may be attributed to the excessive amount of membrane materials in the solution, which causes a large number of particles to accumulate and fail to react properly. Therefore, 1 µL of liposome was preferred as the optimal dosage for constructing LACs. Next, considering the timeliness of the whole experiment, we studied the effects of package incubation time and transcription‐digestion reaction time in LACs on encapsulation efficiency. When the incubation time reached 3 h, a significant increase in MFI was observed (Figure S6B and S6E). Fluorescence increased in a time‐dependent manner, and when the one‐pot reaction reached 4 h, the reaction product increased most significantly (Figure S6C and S6F). Under the optimal conditions, we performed fluorescence kinetic analysis of liposomes containing FAM‐DNA (Figure S7A and S7C). Fluorescent probes without liposomes were set as the control group, while fluorescent probes with liposomes were set as the experimental group. According to Figure S7C, we found that the time required for liposomes to encapsulate DNA probes was remarkably short, and most of the fluorescent probes were encapsulated within just 10 min (red line). This rapid encapsulation resulted in a significant reduction in fluorescence intensity in the solution when compared to the control group without liposomes (black line), and the fluorescence in the solution did not change significantly with the extension of incubation time. These results indicate that fluorescent probes can indeed be effectively encapsulated within liposomes. By calculating the average fluorescence ratio at each time point and the blank group, the corresponding encapsulation efficiency can be obtained to be about 61.16%. Additionally, we also performed fluorescence kinetic analysis of LACs with the total system under optimal conditions (Figure S7B and S7D). The blank group was the total system in the solution without liposomes, and the experimental group was the prepared LACs. Similar to the above results, the one‐pot reaction in the solution reached the highest fluorescence intensity after about 1 h, while the experimental group remained unchanged at a certain level, indicating that the liposomes include almost all the fluorescence reaction products. According to the ratio of the maximum slopes of the two curves obtained in the experimental group and the blank group, the encapsulation efficiency obtained by subtracting this ratio as a whole is calculated to be approximately 84.54%. The above results indicated that the LACs have good encapsulation efficiency and stable performance, which can be utilized for subsequent experiments. By further using NTA to detect the density of two kinds of coatings, LACs (Figure S7E), we found that there were about 4.7 × 10^5^ particles wrapped by fluorescent probes and 4.5 × 10^4^ particles wrapped by the total system on average. According to the above encapsulation efficiency, it can be calculated that the average encapsulated DNA in each LAC is 0.129 pM and 1.878 pM, respectively, and the encapsulated T7 RP enzyme activity is 9.393 × 10^−5^ U.

After a series of characterization experiments, we adopt prepared LACs for testing the efficacy of DOX, DDP, and BLM. As shown in Figure S8A, we selected NTPs as the synthesis switch. After encapsulating other materials, the drugs were added into the solution containing LACs, which caused different degrees of damage to the double‐stranded DNA in LACs. Then, adding NTPs to initiate a one‐pot reaction, the results of flow cytometry showed that with the increase of drug concentration, the representative flow pattern shifted to the left (Figure S8B–D). The corresponding MFI confirmed that the three drugs led to a significant fluorescence decrease (Figure S8E–G), which was consistent with the results in the solution, especially in the DOX groups. Moreover, we observed that the high concentration of DDP and BLM also showed the corresponding MFI gradient; especially, the high concentration of bleomycin almost reached fluorescence saturation, indicating that in the selected time, a higher concentration of antitumor drugs may not lead to too much change in fluorescence. In addition, we observed that under the same concentration, the fluorescence signals of the one‐pot reaction in LACs were significantly lower than those in the solution, which was speculated to be attributed to the fact that LACs could be used as an independent microreactor to enrich a large amount of DNA templates, which increases the probability of drug interaction, thus improving the efficiency of drug testing.

### 2.4 Characterization of CCMACs and Comparison With LACs in Evaluating Antitumor Efficacy

**FIGURE 3:**
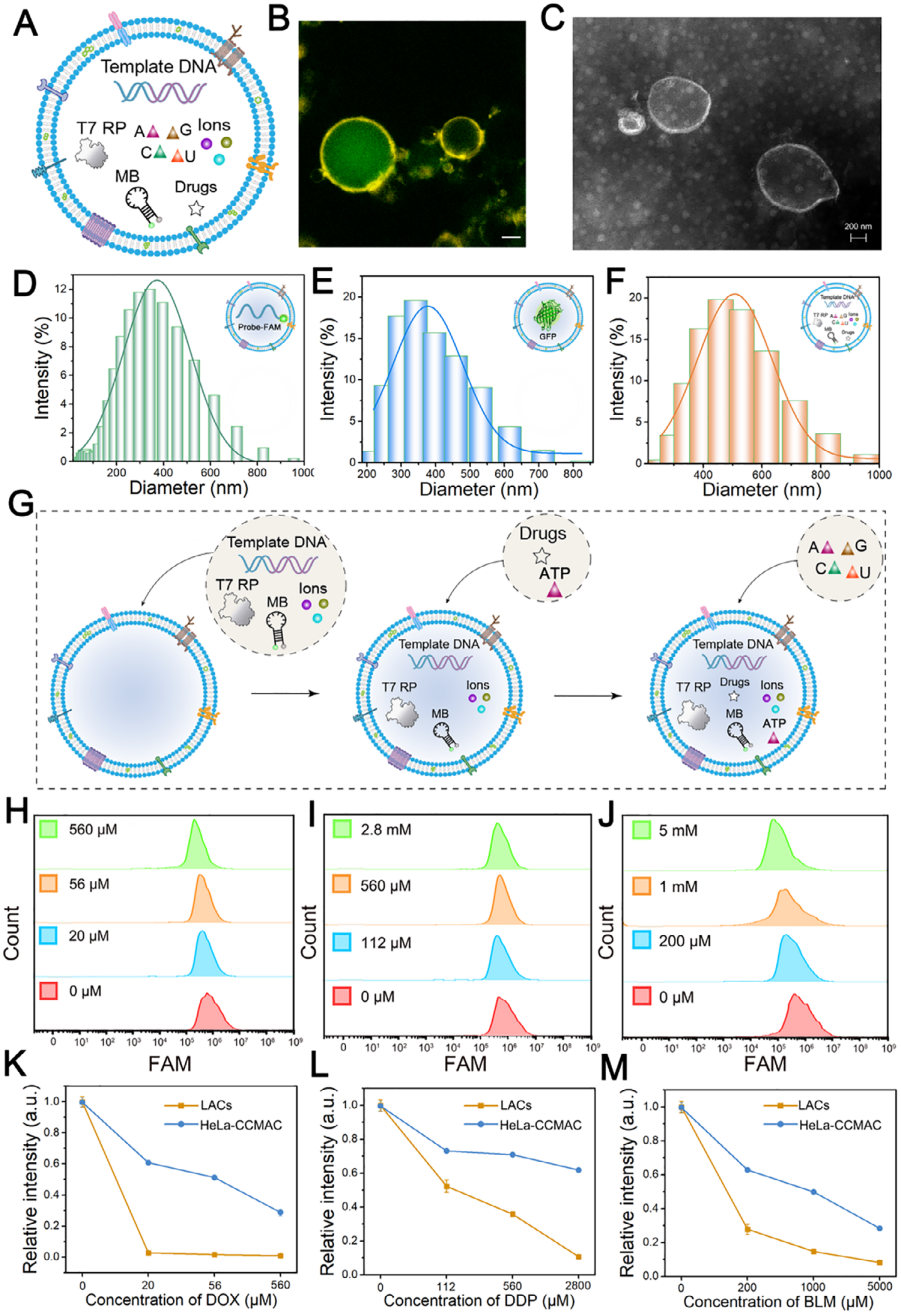
Characterization of CCMAC and its ability to reflect drug efficacy. (A) Schematic diagram of CCMAC construction based on complex biologically derived membrane. (B) Fluorescent microscopy images of OPRS@CCMACs. Scale bars, 200 nm. (C) Transmission electron microscopy images of OPRS@CCMACs. Scale bars, 200 nm. Dynamic light scattering analysis of the template DNA‐FQ@CCMACs (D), GFP@CCMACs (E), OPRS@CCMACs (F). (G) Schematic illustration of antitumor drug sensitivity test performed by OPRS@CCMACs. (H‒J) Representative flow cytometric profiles in OPRS@CCMACs at different concentrations of DOX, DDP, and BLM. (K‒M) Comparison of normalized fluorescence results of LACs and HeLa‐CCMAC for antitumor drug sensitivity evaluation. The fluorescence value was analyzed by FlowJo v10 software. In K‒M, LACs and HeLa‐CCMAC data points represent the average of three independent experiments ± SD, respectively.

After determining that LACs can detect the sensitivity of antitumor drugs, we next evaluated the feasibility of artificial cells derived from real CCMs for sensitivity detection of drugs in situ, so‐called “surrogate testing.” It is well known that the permeability of the membrane to small molecules depends largely on the composition of the membrane [55, 56]. The biomembranes of LACs and CCMACs are based on the phospholipid bilayer, while the components on the CCMACs membrane are more complex, including cholesterol and various transmembrane transporters. Similar to LACs, we first used the purified cell membrane extracted from HeLa cells as membrane material to encapsulate IVT materials of the one‐pot reaction to establish CCMACs (Figure 3A). Then the lipophilic dye Dil was used to stain the CCMACs encapsulating the IVT and digestion system as shown in Figure 3B. Through fluorescence microscopy, we also found that the outer membrane and interior of CCMACs showed orange and green, respectively, indicating that a transcription‐enzyme digestion reaction can also occur in CCMACs. Of note, CCMACs populations also exhibited different fluorescence intensities of FAM at the same incubation time. We speculate that such a change may be due to vesicle aggregation, especially the natural adhesion between tumor‐derived membranes used by CCMACs, or a different level of molecular exchange with the surrounding buffer [26, 53, 54, 57, 58]. Moreover, OPRS@CCMACs were characterized by TEM images (Figure 3C) and DLS (Figure 3F), revealing a predominantly spherical morphology with a size of 500 nm, which was larger than that of LACs. Similarly, to further prove the encapsulation ability of CCMACs, two CCMACs containing FAM‐DNA templates and GFP were prepared in the same way in 1 × PBS. The DLS results showed that the particle size distributions of both CCMACs were approximately 342 nm (Figure 3D,E). Furthermore, fluorescence imaging characterization of CCMACs encapsulated with DNA‐FQ and GFP was conducted, as depicted in Figure S9A and S9B. These results suggest that the volume of one‐pot reaction material encapsulated by tumor cell‐derived membrane is greater than that of individual components.

To ensure the isolated cell membrane is in the correct spatial orientation, we determined the sialic acid content on the cell membrane surface of MCF‐7‐CCMAC and MCF‐7 [59, 60]. Figure S10 shows that the sialic acid content from CCMAC is basically the same as that of real tumor cells, indicating that the CCMACs derived from MCF‐7 have no obvious membrane inversion phenomenon and the correct orientation of the membrane protein of the corresponding source cell has been restored as much as possible, which implies that the membrane protein on these CCMACs can be used for subsequent research. Then, we compared the drug uptake with the same amount of MCF‐7‐CCMAC and its source cells. After making the standard curves of drug characteristic peaks for drug concentration (Figure S11A‒C), we observed that the drugs of randomly selected high concentrations were absorbed by MCF‐7 cells and MCF‐7‐CCMAC to a similar extent, which indicated that these tests could accurately reflect the actual drug resistance effect (Figure S11D‒F).

It has been proved that the MDR mechanism is mainly manifested in the failure of drugs to reach the concentration (or activity) required for treatment in cancer cells, which may be the failure of target drugs to enter targeted cells and/or drug efflux [61, 62], such as p‐glycoprotein (p‐gp/ABCB1) and multi‐drug resistance protein 1 (MRP‐1/ABCC1) with efflux‐promoting effects, which belong to the ATP‐binding cassette transporter superfamily (ABC) [37, 38, 39, 40], and solute carrier family 7 member 11 (SLC7A11/XCT) with uptake‐resisting effects such as in the solute transport protein family (SLC) [41, 42]. We investigated the CCMACs constructed by HeLa cell membranes for the testing of three antitumor drugs. As shown in Figure 3G, similar to Figure S8A, we gradually encapsulated the IVT‐enzyme digestion system, drugs, and ATP into CCMACs and adopted NTPs as the initiation switch. Considering the difference between natural tumor cell membranes and simple phospholipid bilayers, we first studied the effect of ATP as an energy molecule on the membrane transport function of CCMACs (Figure S12). Under the same concentration of drugs and different concentrations of ATP in situ, the proteolysis of ATP on CCMACs membrane provided an energy source for the “pump” to reduce the effect of DOX on the DNA template in CCMACs, thus promoting the generation of fluorescence signals. In the absence or presence of a low concentration of ATP in the solution, high concentrations of DOX were enriched in CCMACs, resulting in inhibition of the IVT system and low fluorescence signals. With the increase of ATP, the DOX efflux gradually increased, leading to a gradual increase in OPRS products and fluorescence signal until it became saturated at 8 mM. Therefore, 8 mM ATP is used as an assisted molecule in subsequent systems for evaluating the effect of drugs. Finally, we adopted CCMACs for testing the same concentration of DOX, DDP, and BLM using flow cytometry. As the concentration of three drugs increased, the flow pattern moved relatively slowly to the left (Figure 3H‒J), which is consistent with the confocal imaging results to visualize the sensitivity test of antitumor drugs in solution (Figure S13). We also compared the normalized MFI of CCMAC and LACs, as shown in Figure 3K‒M. Weaker drug efficacy was presented using CCMACs, especially the significant decrease in drug sensitivity to DOX, which indicated that membrane transporters inhibited the destruction of DNA templates by drugs and tumor‐mimic CCMACs could be used for in situ testing of antitumor drugs.

### 2.5 Exploration of CCMACs Derived From Different Tumor Cell Membranes for Testing of Antitumor Drug Sensitivity

**FIGURE 4:**
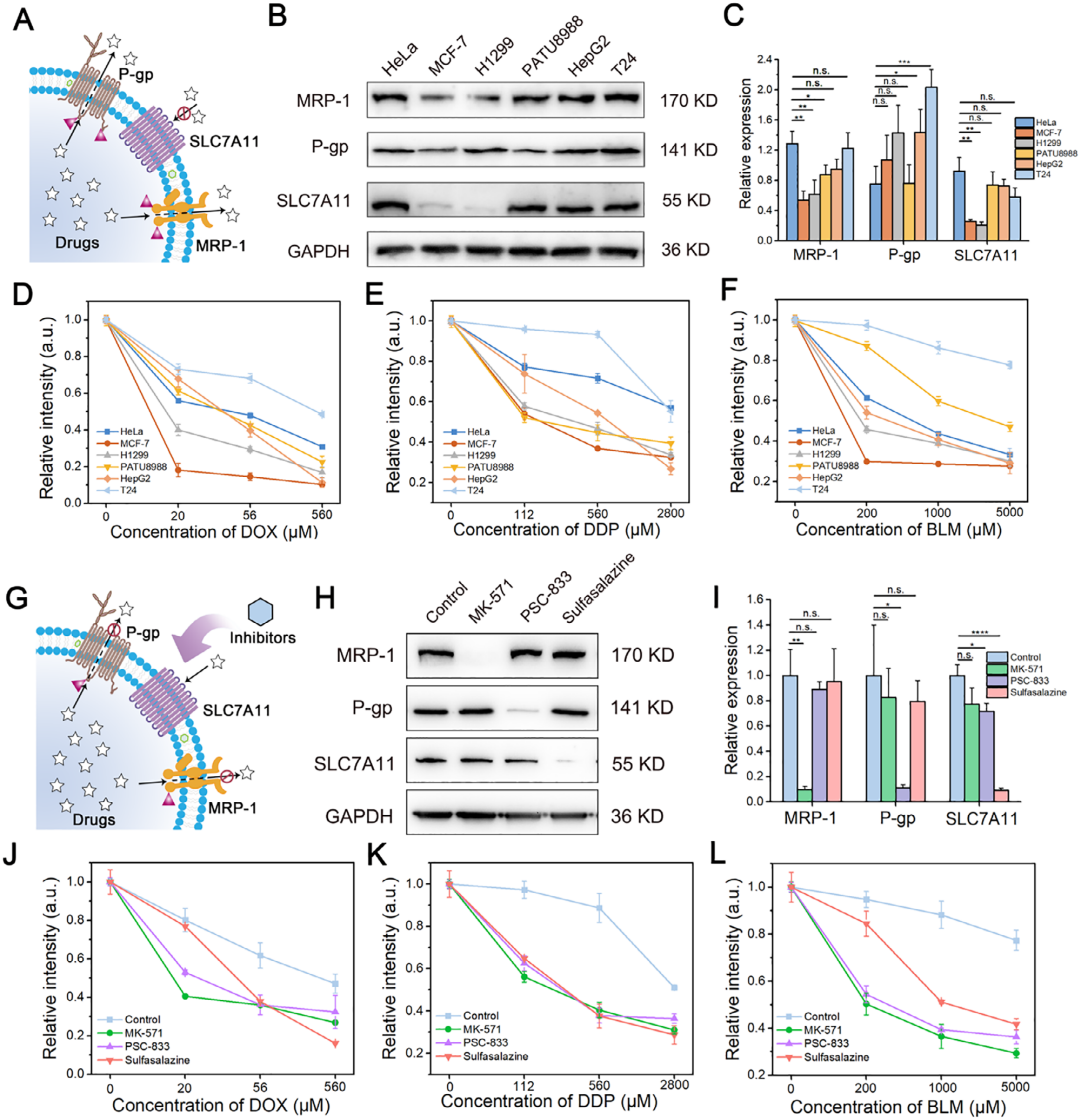
Comparison of CCMAC models from different tumor cell lines in detecting drug efficacy. A) Schematic illustration of inhibition of antitumor drug function due to the presence of multidrug resistance proteins on CCMACs. B‐C) Western blotting analysis of MRP‐1, P‐gp, and SLC7A11 levels in CCMACs derived from HeLa, MCF‐7, H1299, PATU8988, HepG2, and T24. D‐F) Comparison of normalized fluorescence results of HeLa‐CCMAC, MCF‐7‐CCMAC, H1299‐CCMAC, PATU8988‐CCMAC, HepG2‐CCMAC, and T24‐CCMAC for antitumor drug sensitivity evaluation. G) Schematic illustration of the influence of multidrug resistance inhibitors on CCMACs in evaluating the efficacy of antitumor drugs. H‐I) Western blotting analysis of MRP‐1, P‐gp, and SLC7A11 levels in CCMACs derived from T24 treated with MK‐571, PSC‐833, and sulfasalazine, respectively. J‐L) Comparison of normalized fluorescence results of CCMACs derived from T24 treated with MK‐571, PSC‐833, and sulfasalazine for antitumor drug sensitivity evaluation. The fluorescence value was analyzed by FlowJo v10 software. In C‐F and I‐L, each data point represents the average of three independent experiments ± SD. Statistical significance was determined by a two‐tailed student's *t*‐test: *p < 0.05, **p < 0.01, ***p < 0.001, ****p < 0.0001; n.s., not significant (*p >* 0.05).

We further investigated the performance comparison of CCMACs constructed with different tumor cell membranes for the testing of antitumor drugs [63, 64]. First, sodium dodecyl sulfate‐polyacrylamide gel electrophoresis (SDS‐PAGE) and protein staining confirmed that reagent A could separate tumor cell membrane (Figure S14), which ensured that the characteristic proteins inherited from CCM during the preparation of CCMACs were well preserved. We explored the effect of membrane transport resistance proteins on the efficacy of CCMACs model tests (Figure 4A). Here, we randomly selected six representative cell lines of HeLa (human cervical cancer cell), MCF‐7 (human breast cancer cell), H1299 (human lung cancer cell), PATU8988 (human pancreatic cancer cell), HepG2 (human hepatocellular carcinoma cell), and T24 (human bladder transitional cell cancer cell) as the source of derivative membranes of CCMACs. Western blot analysis showed different levels of three resistance proteins in CCM from 6 cancer cells (Figure 4B), which were normalized with the gray analysis results of HeLa, as shown in Figure 4C. We found that the content of MRP‐1 and SLC7A11 on cell membranes of MCF‐7 and H1299 were relatively lower compared with other cells and significantly reduced compared with HeLa cells. The content of P‐gp protein on T‐24 and HepG2 cells was relatively higher compared with other cells and significantly higher than on HeLa cells. Subsequently, the prepared equivalent amounts of CCMACs were adopted to evaluate the effects of three antitumor drugs (Figure 4D‒F, Figures S15, and S16). In general, due to the different permeability of biomembranes and the existence of MDR, the drug resistance of each CCMAC was stronger than that of LAC. Among them, fluorescent signals in CCMACs derived from MCF‐7 and H1299 were lower at different concentrations of three drugs, indicating that relatively more of the drugs could penetrate the membrane to destroy template DNA. On the other hand, it is interesting to note that T24‐CCMAC showed high fluorescence signal, presumably due to the high content of drug‐resistant proteins on the cell membrane of T24. All the above results have displayed that different expression levels of drug resistance proteins in a variety of malignant solid tumors will directly affect the pharmacodynamic evaluation of CCMACs, which demonstrated that our method could be used as a platform for efficacy testing and provide theoretical guidance for personalized treatment of cancer.

Meanwhile, we randomly selected MCF‐7‐CCMAC to test the specific minimum detection limit of three drugs. It is not difficult to infer from Figure 4D‒F that the highest concentration of MCF‐7‐CCMAC in the test of three drugs is almost completely saturated. The normalized MFI showed a good linear relationship with the drugs’ concentrations in the range of 2–14 µM for DOX, 15–80 µM for DDP, and 10–150 µM for BLM (Figure S17). The limits of detection (LOD) were calculated to be 0.35 µM, 7.8 µM, and 9.3 µM, respectively (LOD = 3σ/k, σ is the standard deviation of the blank sample; k is the slope of the fitted curve). In order to further verify the universality of our platform, we independently tested two other antitumor drugs that prevent normal transcription on the same principle as DOX. As shown in Figure S18, the efficacy of epirubicin and daunorubicin can be evaluated by the system in solution and HeLa‐CCMAC, respectively. At the same highest concentration, the inhibitory effect of daunorubicin is below 20%, which is better than that of DOX (Figure 4D), while the inhibitory effects of the same low concentrations of epirubicin on HeLa‐CCMAC are even worse than that of DDP (Figure 4E).

Next, we studied the effect of in situ sensitivity testing following reduction of expression of resistance proteins by resistance protein inhibitors (reversal agents). Considering that the pathways leading to MDR in living cells may be more complicated, we first analyzed the effect of the MDR reversal agent on the physiological activity of tumor cells by the CCK8 colorimetric method (Figure S19). Here, T24 cells with significant resistance were selected as the research subjects. The living T24 cells in the control group were treated without a reversal agent. After incubating with different concentrations of three antitumor drugs, the cell viability of control groups decreased to different extents, but the average survival rate reached 80%. However, the survival state of a few living cells was even better than that of blank cells without reversal agents and drugs, which was presumably attributed to the fact that the expression levels of the affected cell surface resistance proteins were higher than those of the blank cells [65]. After treatment with MK‐571 [66], PSC‐833 [67], and sulfasalazine [68] against each of the three drug‐resistant proteins, the survival rate of T24 cells in the experimental group was higher before the addition of antitumor drugs. Subsequently, it was observed that when different concentrations of DOX (Figure S19A), DDP (Figure S19B), and BLM (Figure S19C) were added, most of the cell viability was significantly reduced, which was related to the fact that the reversal agents reduced the expression of drug‐resistant protein or inhibited the normal function of drug‐resistant protein, indicating that the reversal agents selected had good reversing activity and low cytotoxicity.

Finally, we explored the efficacy of T24 tumor cell membrane‐derived CCMACs after the function of resistance protein inhibitors for the detection of antitumor drugs (Figure 4G). The levels of the three drug‐resistant proteins in situ were analyzed by Western blot (Figure 4H). The gray analysis results of the blank group without inhibitors were normalized as a standard. The levels of the drug‐resistant proteins on the T24 cell membrane were all significantly decreased after the effects of the three reversal agents, while the contents of other proteins were also decreased but could be ignored relative to the blank group (Figure 4I). Therein, the expression of SLC7A11 was most affected by the reversal agent. We performed membrane separation on T24 cells treated with the reversal agents and used the prepared CCMACs to evaluate the effects of three antitumor drugs (Figure 4J‒L, Figure S20). The results showed that after the function of reversal agents, the fluorescence signal decreased significantly with the increase of drug concentration, indicating that the cell resistance of CCMACs decreased to different degrees, and the reversal effect of sulfasalazine was the lowest, which might be attributed to the low content of SLC7A11. Fortunately, the cell survival rate is mostly below 40% compared to the blank group without drugs, indicating that the reversal agents can reduce the cell resistance (Figure S19). Overall, our established CCMACs are allowed to be utilized for testing of drug sensitivity and respond to the regulation of tumor resistance by reversal agents.

For the purpose of comparing the responses of real tumor cells and CCMACs to the three drugs, we chose HeLa cells as a representative model and conducted synchronous tests at the same drug concentrations. According to Figure S21, we observed that HeLa cells exhibited greater drug tolerance compared to HeLa‐CCMAC, possibly attributed to certain drugs targeting genes within the nucleus post‐cell entry and bypassing the DNA template we transfected. In Figure S21C, it is evident that the fluorescence interference stems from residual DOX with red fluorescence in real cells, while DDP and BLM do not exhibit this phenomenon. Additionally, FAM fluorescence decreases as drug concentration rises (Figure S21F, S21I), underscoring the significance of choosing the proportion of gene concentration and drug concentration in the simulation process. Therefore, our findings highlight the advantages of utilizing the artificial cells we developed for in vitro testing to prevent real cells from adhering to the drug. Furthermore, to evaluate the potential value of our platform in clinical applications, we adopted MDA‐MB‐231 (breast cancer cells) and MIA‐PACA‐2 (pancreatic cancer cells) from PDX to construct CCMACs and carried out characterization and drug sensitivity testing, respectively. We observed that the particle sizes of CCMACs were concentrated at 615 nm and 513 nm, respectively, corresponding to fluorescence imaging (Figure S22). Two sources of CCMACs showed high drug resistance (Figure S23), further indicating that our model can be applied in different cell types and used as a data reference.

### 2.6 A Platform for Testing the Drug Efficacy Through 3D Coded Artificial Cells

**FIGURE 5:**
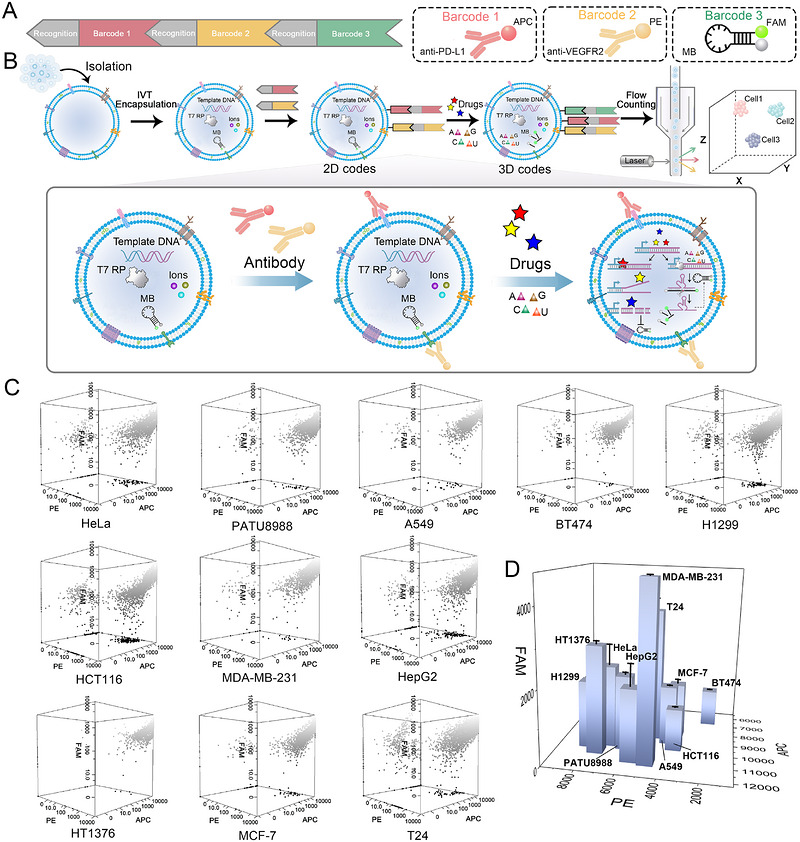
Establishment and application of three‐dimensional pharmacodynamic test artificial cells. A) Schematic diagram of 3D fluorescent coding. B) Schematic illustration of the principle of the 3D‐coded CCMAC platform for detecting drug efficacy. C) Under the treatment of 56 µM DOX, the trichromatically coded 3D scatter plots of CCMACs derived from MDA‐MB‐231, PATU8988, A549, BT474, H1299, HCT116, HeLa, HepG2, HT1376, MCF‐7, and T24 cell membranes. D) 3D bar plot of the mean fluorescence intensities under trichromatic coding. The fluorescence value was analyzed by FlowJo v10 software. In D, each FAM data point represents the average of three independent experiments ± SD.

In order to accurately reflect the clinical samples, it is essential for CCMAC itself, possessing the cell‐specific characteristics, to provide targeted information for the diagnosis and prognosis of specific diseases. Consider endowing CCMAC with extended functions; here we classify the cell‐specific features more accurately by labeling with highly expressed membrane proteins in situ on tumor cells, thus introducing dual recognition information. The corresponding anti‐programmed death ligand‐1 (anti‐PD‐L1) and anti‐vascular endothelial growth factor receptor‐2 (anti‐VEGFR2) were stained by APC and PE, respectively [69, 70], and the cleaving MB generated by IVT inside CCMACs was used as the fluorescence source of 3D coding together (Figure 5A).

After conjugating the CCMACs with two fluorescent antibodies, the third label was obtained for drug action and one‐pot reactions. Subsequently, the trichromatic coding of each CCMAC was simultaneously detected using flow cytometry (Figure 5B). Specifically, CCMACs encapsulated with synthetic materials were sequentially stained by anti‐PD‐L1‐APC and anti‐VEGFR2‐PE, followed by the addition of 56 µM DOX for 2 h. The CCMACs were then washed three times before the addition of NTPs to initiate the one‐pot reaction. The fluorescence spatial distribution of CCMACs from 11 cell membranes after trichromatic coding is illustrated in Figure 5C. Variations in the expression levels of PD‐L1 and VEGFR2, as well as differences in drug resistance among the cells, led to distinct fluorescence distributions among the 11 CCMACs. It is noted that the two kinds of membrane fluorescent antibodies have different MFI results for cell lines from different sources (Figure 5D). Among CCMACs derived from breast cancer, the expression of PD‐L1 on MDA‐MB‐231 is higher than that of MCF‐7 and BT474, while the content of VEGFR2 follows the order of MDA‐MB‐231>MCF‐7>BT474. However, the internal FAM result remains consistent with that of PD‐L1 after treatment with drugs. Conversely, the trichromatic fluorescence encoded by H1299 and A549, both types of lung cancer, exhibit striking differences in their spectral characteristics. Additionally, significant variations exist among different tumor cell lines. We noted that the PD‐L1 content of PATU8988 (pancreatic cancer) and HCT116 (colorectal cancer) is comparable, but the VEGFR2 content of 8988 is nearly double that of HCT116, and the MFI of FAM is also approximately twice as high as that of HCT116.

Furthermore, it is worth noting that the two membrane proteins we selected are related to the efficacy of the drug. From Figure 5D, it is evident that there is a correlation between lower expression levels of membrane proteins and reduced FAM fluorescence, which can be observed in cell lines such as BT474, H1299, and others. We speculate that this may be attributed to the insufficient drug resistance of certain cells, which allows for the easy penetration of drugs into the interior, thereby hindering the initiation of IVT inside CCMACs. In connection with the discussion of Figure 4, our approach allows for the possibility of combining various tumor‐specific target inhibitors with small molecule chemotherapy in the future, which holds promise for significantly enhancing the efficacy of cancer treatment across different tumor types in diverse patient populations.

## 3 Conclusion

In summary, we have developed an artificial cell based on bionic cell membranes and in situ synthetic biology to evaluate the efficacy of different antitumor drugs. By integrating the tumor cell membrane that is the first response of tumor drugs and artificially designed genes that are the direct targets of many tumor drugs, the artificial cell we constructed can respond to different concentrations of antitumor drugs, thus saving time and operating costs. Of note, the membrane‐coated synthetic biological elements here have the advantages of flexibility, convenience, and versatility due to the ease of controlling reaction substrates and conditions in vitro operations. By simulating the reaction of tumor drugs, our constructed artificial cell can identify effective drugs for specific types of tumors faster and more effectively than conventional tumor cells. Considering that efficacy tests conducted at the living cell level are limited by unexpected events that may occur in cancer cells, our established artificial cells can compensate for the lack of both non‐living experiments and living experiments. Therefore, this approach is anticipated to accelerate the screening and development of antitumor drugs while minimizing the reliance on animal testing. Moreover, utilizing artificial cells derived from patients' own cells allows for the evaluation of personalized treatment regimens for specific tumors, offering increased flexibility, accuracy, and controllability. Through in‐depth analysis of the drug sensitivity of artificial cells, different types of chemotherapy drugs and different targeted inhibitors always change according to the patient's condition. This allows us to make more accurate and effective treatments for each patient so as to maximize the treatment effect and reduce the occurrence of side effects. Furthermore, it is allowed to develop an integrated platform (such as microfluidic chips) to design automated analysis equipment or combine deep learning algorithms to calculate the fluorescence signals within artificial cells. These advancements could help eliminate hidden operational variations in the experimental process and overcome the limitations associated with bulk testing technology in the future.

## 4 Experimental Section

### 4.1 Materials and Reagents

All DNA oligonucleotides were synthesized by Hippo Biotechnology Co., Ltd. (Beijing, China) and purified by HPLC. The sequences are shown in Table S1. Iron (II) chloride tetrahydrate (FeCl_2_·4H_2_O) was purchased from Acmec (Shanghai, China), and magnesium chloride hexahydrate (MgCl_2_·6H_2_O) was obtained from Sigma Aldrich (USA). T7 RNA polymerase, ATP, GTP, CTP, and UTP were purchased from ApexBio (APExBIO, Shanghai, China). 5 × loading buffer (containing GelRed nucleic acid dye) was purchased from Generay Biotech Co., Ltd. (Shanghai, China). Doxorubicin (DOX) and sulfasalazine were purchased from Aladdin (Shanghai, China). Cisplatin (DDP), sialidase, and PSC‐833 were obtained from Medchemexpress Co., Ltd. (Shanghai, China). Bleomycin (BLM), MK‐571, Lipo8000 transfection reagent, membrane and cytosol protein extraction kit, phenylmethanesulfonyl fluoride (PMSF), coomassie brilliant blue, polyvinylidene fluoride (PVDF) membrane, Tris‐HCl, and DEPC‐treated water (DNase/RNase free) were obtained from Beyotime Biotechnology (Shanghai, China). Sialic acid assay kit was purchased from Nanjing Jiancheng Bioengineering Institute (Nanjing, China). Cell Counting kit‐8 (CCK‐8) was purchased from Dojindo Laboratories (Shanghai, China). Anti‐GAPDH was purchased from Abclonal (Wuhan, China), and anti‐MRP‐1 was purchased from Abbkine (Shanghai, China); anti‐P‐gp, anti‐SLC7A11, and goat anti‐rabbit immunoglobulin G conjugated to horseradish peroxidase (IgG/HRP) were obtained from Solarbio (Beijing, China). APC‐labeled anti‐PD‐L1 and PE‐labeled anti‐VEGFR2 were purchased from Sino Biological (USA). The BCA protein quantification kit was ordered from Vazyme Biotechnology Co., Ltd. (Nanjing, China). Lipophilic dye Dil was purchased from Sangon Biotechchnology (Shanghai, China). Dulbecco's modified Eagle medium (DMEM), RPMI 1640 medium, fetal bovine serum (FBS), and penicillin‐streptomycin‐neomycin antibiotic mixture were obtained from Thermo Fisher Scientific Co., Ltd. (Shanghai, China). All solutions were prepared with Milli‐Q water (18.2 MΩ cm^−1^) from a Milli‐Q purification system (Millipore, Milford, MA, USA).

### 4.2 Cell Culture

HeLa (human cervical cancer cell), MCF‐7 (human breast cancer cell), H1299 (human lung cancer cell), PATU8988 (Human pancreatic cancer cell), HepG2 (human hepatocellular carcinoma cell), and T24 (human bladder transitional cell cancer cell) cell lines were purchased from the Institute of Biochemistry and Cell Biology of the Chinese Academy of Sciences (Shanghai, China). MDA‐MB‐231 (breast cancer cells) and MIA‐PACA‐2 (pancreatic cancer cells) from PDX were obtained from the Suzhou Institute of Materia Medica. HeLa, MCF‐7, and HepG2 cells were grown in DMEM with the addition of 10% FBS and 1% penicillin‐streptomycin‐neomycin antibiotic mixture in a humidified atmosphere containing 5% CO_2_ at 37°C. H1299, PATU8988, and T24 cells were grown in RPMI 1640 medium containing 10% FBS and 1% antibiotic mixture under the same culture conditions. These cells were collected at the end of the log phase for the following experiments. In addition, in order to obtain the resistance‐reversing cell lines cultured by T24 cell lines, we first cultured the T24 cell line in normal medium for 24 h, then added probenecid with a final concentration of 0.25 mM, cyclosporine A of 1 µM, or sulfadiazine of 0.25 mM, respectively, incubated for 72 h at 37°C and 5% CO_2_ in an incubator; and collected them with cell scrapers.

### 4.3 In Vitro Transcription (IVT) and Ribozyme Cleavage Reaction

Ribozyme was synthesized using the IVT method and ribozyme‐mediated cleavage reaction as described previously [65, 71]. In short, the DNA template was formed through hybridization of the sense strand and antisense strand that were annealed at 95°C for 5 min and then quickly cooled to room temperature together. For step reaction, 100 nM DNA template was incubated with different concentrations of antitumor drugs (Dox: 0, 2, 10, 20 µM; DDP: 0, 5, 25, 50, 100 µM; BLM: 0, 20, 100, 200 µM) for 2 h. Then the HyperScribe T7 high‐yield RNA synthesis kit was used for IVT. The above reaction system was mixed with 2 mM adenosine triphosphate (ATP), 2 mM guanosine triphosphate (GTP), 2 mM cytidine triphosphate (CTP), 2 mM uridine triphosphate (UTP), 2 µL 10 × T7 reaction buffer, and 5 U T7 RNA polymerase (T7 RP) in a final volume of 20 µL and incubated at 37°C for 4 h to synthetic ribozyme, followed by the removal of template DNA using 2 U DNase I. The substrate as a molecular beacon (MB) was modified with a FAM fluorophore and quenching group. Ribozyme cleavage reaction was conducted by adding 500 nM MB, ribozyme, and 1 × cleavage buffer (50 mM Tris‐HCl, pH 7.5, 20 mM MgCl_2_) in a final volume of 60 µL. The mixture was incubated at 37°C for 4 h. On the other hand, a one‐pot reaction combined IVT and a ribozyme cleavage reaction. In brief, 100 nM DNA template after incubating with antitumor drugs (Dox: 0, 2, 10, 20 µM; DDP: 0, 5, 25, 50, 100 µM; BLM: 0, 20, 100, 200 µM; Daunorubicin: 0, 2, 6, 10, 20 µM; Epirubicin: 0, 2, 10, 50, 150 µM) was mixed with 2 mM ATP, 2 mM GTP, 2 mM CTP, 2 mM UTP, 1 × T7 reaction buffer, 5 U T7 RP, 500 nM MB, and 1 × cleavage buffer in a final volume of 60 µL. After incubation at 37°C for 4 h, the fluorescence intensity was measured by the F‐7000 fluorescence spectrometer (Hitachi, Japan) with an excitation wavelength of 520 nm and an emission wavelength of 490 nm.

### 4.4 PAGE

15% non‐denaturing PAGE was used to validate the effect of drugs in the solution on the transcription of the DNA template. In brief, transcription products (8 µL) together with 5 × loading buffer (2 µL) containing GelRed were loaded onto a 15% gel. Electrophoresis separation was then run at 120 V for 60 min in 1 × TBE buffer. An image of the gel was obtained using a GelDoc XR imaging system (Bio‐Rad, USA).

### 4.5 Preparation of CCM Fragments

Referring to the previous literature [72], we further optimized the membrane preparation process of the membrane and cytosol protein extraction kit. To prepare CCM fragments, HeLa, MCF‐7, H1299, PATU8988, HepG2, and T24 cells were cultured into a confluent monolayer and collected by using cell scrapers and then counted with an automated cell counter (Bio‐Rad, US). Next, the cells were centrifuged at 1000 rpm for 5 min, followed by washing with cold 1 × PBS. The centrifugation and washing were repeated three times. After that, the collected cells were suspended in membrane protein extraction reagent A containing 1 mM PMSF, mixed, and incubated in an ice bath for 15 min. The obtained cells were cycled three times at 37°C and liquid nitrogen with an interval of 3 min, repeatedly frozen and thawed to extract cell membrane. The resulting solution was then sonicated on ice using a water‐bath sonicator for 10 min and centrifuged at 700 g at 4°C for 10 min. The supernatant was then carefully collected and centrifuged at 14,000 g for an additional 30 min at 4°C. Finally, the precipitate was collected as purified cell membrane fragments, and the supernatant was saved as plasma proteins, both stored at ‐80°C for subsequent experiments.

### 4.6 Western Blotting

Protein lysates were obtained according to the protocol of reagent B in the membrane and cytosol protein extraction kit [73], and the concentration of proteins was detected by the BCA protein assay kit. After that, the extracted cells were mixed with 5 × loading buffer at a volume ratio of 1:4 and boiled at 95°C for 10 min before use. Equal amounts of membrane proteins and equal amounts of plasma proteins of HeLa, T24, MCF‐7, H1299, PATU8988, and HepG2 cells were separated on 10% sodium dodecyl SDS‐PAGE; the gel was imaged with Coomassie blue ready‐to‐use, and at the appropriate time the imaging was stopped and decolorized with water. Afterward, the samples were imaged on the BioRad ChemiDoc MP gel imaging system. Similarly, equal amounts of CCM proteins from the fragments were separated on 7.5% SDS‐PAGE and transferred to PVDF membranes. After being blocked with 5% skimmed milk, the PVDF membranes were incubated overnight at 4°C with primary antibodies against MRP‐1, P‐gp, SLC7A11, and GAPDH. Afterward, the PVDF membranes were washed three times and incubated at room temperature for 2 h with goat anti‐rabbit lgG/HRP. Finally, the membranes were visualized by enhanced chemiluminescence kit and visualized on the Tanon‐5200 Chemiluminescent Imaging System (Tanon Science & Technology). ImageJ software was used to analyze relative protein expression by calculating the grayscale blots.

### 4.7 Construction of Liposome‐Based and CCM‐Based Artificial Cells

Materials such as DNA‐FQ, GFP, or OPRS were mixed with 1 µL Lipo8000 transfection reagent and incubated at 37°C for 3 h to construct liposome‐based artificial cells (LACs). Considering that the liposomes we used could not be centrifuged at high speed, LACs were stored at 4°C for later use after low‐speed centrifugation to remove the uncoated mixture. In order to prepare CCM‐based artificial cells (CCMACs), we mixed the same synthetic components as LACs into 5 µL CCMs, exposed the mixture to ultrasound for 3 min in the mode of 3 s duty cycle and 2 s stop cycle, and then incubated at 37°C for 3 h to construct complete CCMACs. After high‐speed centrifugation at 4°C, 10,000 g for 1 min to remove the non‐embedded substances, the obtained CCMAC precipitate was re‐suspended in PBS. The artificial cells formed above are incubated at 37°C or stored at 4°C for later use by adding drugs and NTPs.

### 4.8 Characterization of Artificial Cells

For circular dichroism (CD) analysis, 1 µM template double‐stranded DNA was incubated with 10 µM DOX, 100 µM DDP, and 100 µM BLM at 37°C for 2 h, including the blank control group, and 200 µL of each sample was added to the sample pool for testing with a multifunctional circular dichroism spectrometer (Jasco, China). For dynamic laser light scattering (DLS), the sizes of LACs and CCMACs, respectively coated with template DNA‐FQ, GFP, and a one‐pot reaction system (OPRS), were measured by using a ZETASIZER 3000HS instrument (Malvern Instruments Ltd., UK). For nanoparticle tracking analysis (NTA), the number of LACs coated by template DNA‐FQ and OPRS was detected by using a nanoparticle tracker (Particle Metrix, Germany). For fluorescent microscopy observation, LACs were first incubated for 30 min at 37°C, and CCMACs were prepared by ultrasonic method. After that, they were stained with DiI (30 µM) for 30 min at 37°C, and CCMACs were purified with a 100 KD ultrafiltration centrifuge tube to remove free dyes. Fluorescence images were taken by Zeiss Axio Imager M2 fluorescent microscopy (Zeiss, Germany) equipped with 488 nm and 565 nm lasers to excite FAM and DiI, respectively. Additionally, the fluorescence gradient of drug concentration in HeLa‐CCMACs and the characterization of MDA‐MB‐231‐CCMAC and MIA‐PACA‐2‐CCMAC were conducted under a confocal laser scanning microscope (CLSM) (Leica TCS SP8 STED 3X). For transmission electron microscopy (TEM), OPRS@LACs and OPRS@CCMACs were characterized using a Hitachi H‐7650 transmission electron microscope (Tokyo, Japan).

In order to characterize the membrane orientation of CCMACs after encapsulating the system, we selected an equal amount of MCF‐7‐CCMAC and its source cells (volume ratio is 2:1) after quantifying membrane proteins in a BCA kit, with OPRS@LACs as a negative control and the simple system as a blank control, and incubated sialic acid on the membrane surface for 2 h at room temperature with 20 U sialidase to hydrolyze into the solution, then centrifuged at 14,000 g for 30 min and collected supernatant and detected sialic acid content according to sialic acid colorimetric kit instructions.

In the process of exploring the drug uptake by CCMACs and real cells, we utilized the membrane derived from MCF‐7 cells to make OPRS@MCF‐7‐CCMAC and incubated it with the equal amount of MCF‐7 living cells at 37°C for 2 h to wait for the end of drug uptake, then centrifuged it at 14,000 g for 30 min, and took out the supernatant to test the ultraviolet‐visible spectrum of the remaining drugs and calculated the concentration of the remaining drugs by using the corresponding standard curve.

### 4.9 Evaluation of Artificial Cell Encapsulation

In order to test the encapsulation efficiency of template DNA‐FQ@LACs (E_1_) for DNA strand, the fluorescence intensity of the solution before wrapping and after incubation for different times was recorded by fluorescence spectrometer, and the average encapsulation efficiency was taken as the final data after the fluorescence reached the plateau period. The formula was as follows:

$$E_{1}\left(\%\right)=\left[\left(\frac{F0-F1}{F0}+\frac{F0-F2}{F0}+\frac{F0-F3}{F0}\right)/3\right]\times 100$$

where F0 is the fluorescence intensity without LAC coating at all, and F1, F2, and F3 are the fluorescence intensities of the solution after incubation for a period of time and reaching the maximum encapsulation.

In order to test the encapsulation efficiency of OPRS@LACs (E2) for the total system, the fluorescence dynamics records the fluorescence intensity of the encapsulated system and the completely uncoated reaction solution, and the ratio of the slope (K_max_) where the fluorescence rises fastest is taken as the final data. The formula is as follows:

$$E_{2}\left(\%\right)=\left[1-\left(F_{s}-F_{s-1}\right)/\left(F_{t}-F_{t-1}\right)\right]\times 100\%$$

where F_t_ and F_t‐1_ are the adjacent fluorescence intensities where the fluorescence of the uncoated system rises fastest, the corresponding K_max_ = F—F_t‐1_. F_s_ and F_s‐1_ are the adjacent fluorescence intensities where the reaction fluorescence of the coated system rises fastest; the corresponding K_max_ = F—F_s‐1_.

For the calculation of the average package materials of two kinds of LACs, the density of LACs (ρ, particles/mL) is measured by NTA, and the formula is as follows:

$$C_{/\mathrm{paticle}}=\frac{C\times E}{\left(\frac{\rho }{1000}\right)\times 60}$$

where C is the total concentration of materials in the system, E is the corresponding encapsulation efficiency, and ρ is the density (particles/mL) of the corresponding LACs.

### 4.10 IVT and Ribozyme Cleavage Reaction With LACs/CCMACs

Firstly, we used the ultrasonic method to package 100 nM template DNA, 5 U T7 RP, 500 nM molecular beacon (MB), 1 × T7 reaction buffer and 1 × cleavage buffer, into 1 µL LACs/5 µL CCMACs at 37°C, supplemented to 60 µL with PBS. Then different concentrations of antitumor drugs (DOX, 0, 20, 56, 560 µM; DDP, 0, 112, 560 µM, 2.8 mM; BLM, 0, 200 µM, 1 mM, 5 mM; Daunorubicin: 0, 20, 56, 560 µM; Epirubicin: 0, 112, 560 µM, 2.8 mM) was added to LACs/CCMACs by the free diffusion characteristics of small molecules. Under the influence of drug‐resistant proteins on CCMACs membrane, the actual concentration of drugs acting on the internal DNA template decreased. After incubating with drugs at 37°C for 2 h, NTPs: 2 mM ATP, 2 mM GTP, 2 mM CTP, and 2 mM UTP required for transcription were added to form complete OPRS@LACs/CCMACs. Finally, a one‐pot transcription‐ribozyme cleavage reaction was carried out at 37°C for 4 h, and 240 µL PBS was added for dilution for flow counting and fluorescence determination.

### 4.11 Flow Cytometric Analysis

Artificial cells were diluted in 1 × PBS before analysis. Flow cytometry was conducted using a CytoFLEX Flow Cytometer (Beckman Coulter, USA) with a flow rate setting of “Fast” and voltage values of 30 V SSC‐A, FSC‐A, and 1 V FITC (fluorescein isothiocyanate). For each run, at least 1000 gated events to 5000 total events were recorded with a gating strategy that took into account only fractions containing LACs or CCMACs. And ensure that the number of artificial cell particles effectively recorded in each experiment is equal. For the experimental gating strategy, the “nonfluorescent template DNA (sense strand)” sample was used as a negative controls to determine the FITC‐positive events to create a new gate. Data were analyzed with FlowJo v10 software (FlowJo LLC).

### 4.12 Fluorescence Kinetic Analysis

A one‐pot reaction system in a final volume of 30 µL was performed on a CFX96 Touch Deep Well real‐time system (Bio‐Rad, USA). The mixture was incubated at 37°C for 240 cycles (each cycle was set for 1 min), and FAM fluorescence by ribozyme cleavage reaction in solution was monitored in real time.

### 4.13 Cell Viability Assay

The cell Counting kit‐8 (CCK‐8) assay was applied to detect cell viability as previously described [74]. Briefly, cells were seeded onto 96‐well plates (100 µL/well) at a density of 1 × 10^4^ per well. The next day, cells were treated with three resistance protein inhibitors (50 µM MK‐571, 40 µM PSC‐833, or 0.25 mM sulfasalazine, respectively) for 72 h with normal culture conditions. Subsequently, three antitumor drugs with different concentrations (DOX: 0, 20, 56, 560 µM; DDP: 0, 112, 560 µM, 2.8 mM; BLM: 0, 200 µM, 1 mM, 5 mM) were incubated with three drug resistance reversal cell lines for 2 h in the same environment. Finally, cells were incubated with 10 µL CCK‐8 reagent for 2 h at 37°C, 5% CO_2_ in an incubator. The absorbance at a wavelength of 450 nm was determined using a SpectraMax iD5 Multi‐Mode Microplate Reader (Molecular Devices, USA).

### 4.14 Transfection

Cells were transfected with synthetic DNA templates, MB, and T7 RNA polymerase using Liposome 8000 transfection reagent according to the manufacturer's procedures [75]. More specifically, the contents of the DNA template, MB, T7 RP, and liposome in each 6 cm cell culture dish were 5 µg, 25 µg, 200 U and 8 µL, respectively, and the groups without T7 RP transfection and DNA template transfection were set as the blank group and control group. The two stages of transfection of oligonucleotide and transfection of T7 RP each took 4 h in the cell incubator. Then, the cells were washed 3 times with DMEM medium with 10% FBS before inducing the in vivo transcription‐restriction enzyme digestion reaction. For verifying the feasibility of testing drug efficacy in vivo cells, different final concentrations of drug (DOX: 0, 1.2, 3.3, 32.5 µM; DDP: 0, 6.5, 32.5 µM, 162.4 µM; BLM: 0, 11.6 µM, 58 µM, 290 µM) were added after transfection and washing and then incubated for 2 h, followed by washing three times. It is necessary to avoid light during drug stimulation and transcription‐enzyme digestion reactions in vivo.

### 4.15 Construction of 3D Coded Artificial Cells

For each selected tumor cell line (MDA‐MB‐231, PATU8988, A549, BT474, H1299, HCT116, HeLa, HepG2, HT1376, MCF‐7, T24), the cell membrane was peeled off, which was used to encapsulate a one‐pot system according to the above steps. Then, 2 µL anti‐PD‐L1‐APC and anti‐VEGFR2‐PE (5 µL for 10^4^ particles) were added, respectively, and incubated with CCMACs in the dark at 4°C for 0.5 h. After incubation with 56 µM DOX for 2 h, NTPs were added to initiate the internal one‐pot reaction. The reaction conditions are the same as the above‐mentioned flow cytometric experiment. The CytoFLEX Flow Cytometer (Beckman Coulter, USA) was used to count 5000 particles in three repeated experiments of each CCMAC, and the data processing of coding fluorescence was carried out by FlowJo v10 software (FlowJo LLC). Including the generation of multi‐layer original spectrograms and corresponding average fluorescence data statistics about FITC‐A, PE‐A, and APC‐A, as well as three‐dimensional point graphs generated in 3D coding CCMACs, etc.

### 4.16 Statistical Analysis

All the experiments were repeated at least three times, and the obtained data were expressed as means ± standard deviation. A two‐tailed student's t‐test was used to evaluate statistical mean differences between two groups. All statistical analyses were performed using the GraphPad software (Prism 9.5), and *p <* 0.05 was regarded as statistically significant.

## Author Contributions

C.G. and R.Z. contributed equally to this work. C.F. and G.C. were associated with concept design and guidance. C.G. and R.Z. performed experiments, data analysis, and manuscript writing. C.F. and G.C. revised and edited the manuscript. Z.Z., P.S., Y.M., C.M., and Y.M. performed an antitumor drug study and tumor cells collection. Q.M. and G.C. take responsibility for research supervision and funding acquisition. All authors approved the submission of the final manuscript.

## Conflicts of Interest

The authors declare no conflicts of interest.

## Supporting information




**Supporting File**: exp270177‐sup‐0001‐SuppMat.pdf.

## Data Availability

The data that support the findings of this study are available from the corresponding author upon reasonable request.

## References

[1] H. Niu , M. Xu , S. Li , et al., “High‐Performance Liquid Chromatography (HPLC) Quantification of Liposome‐Delivered Doxorubicin in Arthritic Joints of Collagen‐Induced Arthritis Rats,” Medical Science Monitor Basic Research 23 (2017): 150–158, 10.12659/MSMBR.904103. 28408733 PMC5400028

[2] Z. Zhang , X. Zhang , and S. Zhang , “Heart‐Cut Capillary Electrophoresis for Drug Analysis in Mouse Blood With Electrochemical Detection,” Analytical Biochemistry 387 (2009): 171–177, 10.1016/j.ab.2009.01.026. 19454242

[3] T. Hayon , A. Dvilansky , O. Shpilberg , and I. Nathan , “Appraisal of the MTT‐Based Assay as a Useful Tool for Predicting Drug Chemosensitivity in Leukemia,” Leukemia & Lymphoma 44 (2003): 1957–1962, 10.1080/1042819031000116607. 14738150

[4] C. M. Kurbacher and I. A. Cree , “Chemosensitivity Testing Using Microplate Adenosine Triphosphate‐Based Luminescence Measurements,” Methods in Molecular Medicine 110 (2005): 101.. 15901931 10.1385/1-59259-869-2:101

[5] L. Huang , B. Bockorny , I. Paul , et al., “PDX‐Derived Organoids Model in Vivo Drug Response and Secrete Biomarkers,” JCI Insight 5 (2020): e135544, 10.1172/jci.insight.135544. 32990680 PMC7710298

[6] L. A. Villarruel Mendoza , N. A. Scilletta , M. G. Bellino , M. F. Desimone , and P. N. Catalano , “Recent Advances in Micro‐Electro‐Mechanical Devices for Controlled Drug Release Applications,” Frontiers in Bioengineering and Biotechnology 8 (2020): 827, 10.3389/fbioe.2020.00827. 32850709 PMC7405504

[7] R. Agius , M. Parviz , and C. U. Niemann , “Artificial Intelligence Models in Chronic Lymphocytic Leukemia—Recommendations toward State‐of‐the‐Art,” Leukemia & Lymphoma 63 (2022): 265–278, 10.1080/10428194.2021.1973672. 34612160

[8] D. M. Kurtz , M. S. Esfahani , F. Scherer , et al., “Dynamic Risk Profiling Using Serial Tumor Biomarkers for Personalized Outcome Prediction,” Cell 178 (2019): 699–713.e19, 10.1016/j.cell.2019.06.011. 31280963 PMC7380118

[9] F. De Pretis , M. van Gils , and M. M. Forsberg , “A Smart Hospital‐Driven Approach to Precision Pharmacovigilance,” Trends in Pharmacological Sciences 43 (2022): 473–481, 10.1016/j.tips.2022.03.009. 35490032

[10] G. Vlachogiannis , S. Hedayat , A. Vatsiou , et al., “Patient‐Derived Organoids Model Treatment Response of Metastatic Gastrointestinal Cancers,” Science 359 (2018): 920–926, 10.1126/science.aao2774. 29472484 PMC6112415

[11] X. Chen , R. Deng , D. Su , et al., “Visual Genetic Typing of Glioma Using Proximity‐Anchored in Situ Spectral Coding Amplification,” Exploration (Beijing) 3 (2023): 20220175, 10.1002/EXP.20220175. 37933281 PMC10582607

[12] S. Ye , J. W. B. Boeter , M. Mihajlovic , et al., “A Chemically Defined Hydrogel for Human Liver Organoid Culture,” Advanced Functional Materials 30 (2020): 2000893, 10.1002/adfm.202000893. 34658689 PMC7611838

[13] K. Kretzschmar , “Cancer Research Using Organoid Technology,” Journal of Molecular Medicine (Berlin) 99 (2021): 501–515, 10.1007/s00109-020-01990-z. PMC8026469 33057820

[14] A. Bhaduri , M. G. Andrews , W. Mancia Leon , et al., “Cell Stress in Cortical Organoids Impairs Molecular Subtype Specification,” Nature 578 (2020): 142–148, 10.1038/s41586-020-1962-0. 31996853 PMC7433012

[15] M. Huch , J. A. Knoblich , M. P. Lutolf , and A. Martinez‐Arias , “The Hope and the Hype of Organoid Research,” Development 144 (2017): 938–941, 10.1242/dev.150201. 28292837

[16] H. Clevers , “Advances in Organoid Technology: Hans Clevers, Madeline Lancaster, and Takanori Takebe,” Cell Stem Cell 20 (2017): 759–762.

[17] M. Mimee , P. Nadeau , A. Hayward , et al., “An Ingestible Bacterial‐Electronic System to Monitor Gastrointestinal Health,” Science 360 (2018): 915–918, 10.1126/science.aas9315. 29798884 PMC6430580

[18] W. Jiang , Z. Wu , Z. Gao , et al., “Artificial Cells: Past, Present and Future,” ACS Nano 16 (2022): 15705–15733, 10.1021/acsnano.2c06104. 36226996

[19] M. A. Boyd , W. Thavarajah , J. B. Lucks , and N. P. Kamat , “Robust and Tunable Performance of a Cell‐Free Biosensor Encapsulated in Lipid Vesicles,” Science Advances 9 (2023): eadd6605, 10.1126/sciadv.add6605. 36598992 PMC9812392

[20] N. Deng , M. A. Vibhute , L. Zheng , H. Zhao , M. Yelleswarapu , and W. T. S. Huck , “Macromolecularly Crowded Protocells from Reversibly Shrinking Monodisperse Liposomes,” Journal of the American Chemical Society 140 (2018): 7399–7402, 10.1021/jacs.8b03123. 29870243 PMC6016064

[21] M. Chang , H. Ariyama , W. T. S. Huck , and N. Deng , “Division in Synthetic Cells,” Chemical Society Reviews 52 (2023): 3307–3325, 10.1039/D2CS00985D. 37066696

[22] Y. Fu , Y. Hu , T. Lin , et al., “Constructing Artificial Gap Junctions to Mediate Intercellular Signal and Mass Transport,” Nature Chemistry 16 (2024): 1418–1426, 10.1038/s41557-024-01519-8. 38658798

[23] J. A. Peruzzi , M. L. Jacobs , T. Q. Vu , K. S. Wang , and N. P. Kamat , “Barcoding Biological Reactions with DNA‐Functionalized Vesicles,” Angewandte Chemie 58 (2019): 18683–18690, 10.1002/anie.201911544. 31596992 PMC6901749

[24] C. Xu , S. Hu , and X. Chen , “Artificial Cells: From Basic Science to Applications,” Materials Today 19 (2016): 516–532, 10.1016/j.mattod.2016.02.020. 28077925 PMC5222523

[25] Z. C. Liu and T. M. Swi Chang , “Artificial Cell Microencapsulated Stem Cells in Regenerative Medicine, Tissue Engineering and Cell Therapy,” Advances in Experimental Medicine and Biology 670 (2010): 68–79.. 20384219 10.1007/978-1-4419-5786-3_7 PMC3518469

[26] M. Dwidar , Y. Seike , S. Kobori , C. Whitaker , T. Matsuura , and Y. Yokobayashi , “Programmable Artificial Cells Using Histamine‐Responsive Synthetic Riboswitch,” Journal of the American Chemical Society 141 (2019): 11103–11114, 10.1021/jacs.9b03300. 31241330

[27] N. A. Bakh , A. B. Cortinas , M. A. Weiss , et al., “Glucose‐Responsive Insulin by Molecular and Physical Design,” Nature Chemistry 9 (2017): 937–944, 10.1038/nchem.2857. 28937662

[28] J. M. Thomas , M. S. Friddin , O. Ces , and Y. Elani , “Programming Membrane Permeability Using Integrated Membrane Pores and Blockers as Molecular Regulators,” Chemical Communications 53 (2017): 12282–12285, 10.1039/C7CC05423H. 29091084

[29] W. Gao and L. Zhang , “Coating Nanoparticles With Cell Membranes for Targeted Drug Delivery,” Journal of Drug Targeting 23 (2015): 619–626, 10.3109/1061186X.2015.1052074. 26453159

[30] D. Matyszewska , “The Influence of Charge and Lipophilicity of Daunorubicin and Idarubicin on Their Penetration of Model Biological Membranes—Langmuir Monolayer and Electrochemical Studies,” BBA‐Biomembranes 1862 (2020): 183104, 10.1016/j.bbamem.2019.183104. 31672546

[31] M. Zaborowska , D. Dziubak , P. Fontaine , and D. Matyszewska , “Influence of Lipophilicity of Anthracyclines on the Interactions with Cholesterol in the Model Cell Membranes—Langmuir Monolayer and SEIRAS Studies,” Colloids and Surfaces: B, Biointerfaces 211 (2022): 112297, 10.1016/j.colsurfb.2021.112297. 34953365

[32] Y. Yu , L. Zhang , Z. Qin , J. Karges , H. Xiao , and X. Su , “Unraveling and Overcoming Platinum Drug‐Resistant Cancer Tumors with DNA Nanostructures,” Advanced Functional Materials 33 (2023): 2208797, 10.1002/adfm.202208797.

[33] B. Yu , Y. Wang , T. Bing , et al., “Platinum Prodrug Nanoparticles With COX‐2 Inhibition Amplify Pyroptosis for Enhanced Chemotherapy and Immune Activation of Pancreatic Cancer,” Advanced Materials 36 (2024): 2310456, 10.1002/adma.202310456. 38092007

[34] E. Mugiyanto , W. Adikusuma , L. M. Irham , W. Huang , W. Chang , and C. Kuo , “Integrated Genomic Analysis to Identify Druggable Targets for Pancreatic Cancer,” Frontiers in Oncology 12 (2022): 989077, 10.3389/fonc.2022.989077. 36531045 PMC9752886

[35] X. Zhen , P. Cheng , and K. Pu , “Recent Advances in Cell Membrane–Camouflaged Nanoparticles for Cancer Phototherapy,” Small 15 (2019): e1804105, 10.1002/smll.201804105. 30457701

[36] M. Z. Alyami , S. K. Alsaiari , Y. Li , et al., “Cell‐Type‐Specific CRISPR/Cas9 Delivery by Biomimetic Metal Organic Frameworks,” Journal of the American Chemical Society 142 (2020): 1715–1720, 10.1021/jacs.9b11638. 31931564

[37] R. W. Robey , K. M. Pluchino , M. D. Hall , A. T. Fojo , S. E. Bates , and M. M. Gottesman , “Revisiting the Role of ABC Transporters in Multidrug‐Resistant Cancer,” Nature Reviews Cancer 18 (2018): 452–464, 10.1038/s41568-018-0005-8. 29643473 PMC6622180

[38] T. Tomono , M. Kajita , K. Yano , and T. Ogihara , “Adenovirus Vector Infection of Non‐Small‐Cell Lung Cancer Cells Is a Trigger for Multi‐Drug Resistance Mediated by P‐glycoprotein,” Biochemical and Biophysical Research Communications 476 (2016): 183–187, 10.1016/j.bbrc.2016.05.070. 27286705

[39] M. Banerjee , G. Kaur , B. D. Whitlock , M. W. Carew , X. C. Le , and E. M. Leslie , “Multidrug Resistance Protein 1 (MRP1/ABCC1)‐Mediated Cellular Protection and Transport of Methylated Arsenic Metabolites Differs Between Human Cell Lines,” Drug Metabolism and Disposition 46 (2018): 1096–1105, 10.1124/dmd.117.079640. 29752257

[40] K. M. Hanssen , M. Haber , and J. I. Fletcher , “Targeting Multidrug Resistance‐Associated Protein 1 (MRP1)‐Expressing Cancers: Beyond Pharmacological Inhibition,” Drug Resistance Updates 59 (2021): 100795, 10.1016/j.drup.2021.100795. 34983733

[41] X. Liu , “SLC Family Transporters,” Advances in Experimental Medicine and Biology 1141 (2019): 101–202.. 31571165 10.1007/978-981-13-7647-4_3

[42] W. Lin , C. Wang , G. Liu , et al., “SLC7A11/xCT in Cancer: Biological Functions and Therapeutic Implications,” American Journal of Cancer Research 10 (2020): 3106. 33163260 PMC7642655

[43] Y. Guo , Z. Wang , X. Shi , and M. Shen , “Engineered Cancer Cell Membranes: An Emerging Agent for Efficient Cancer Theranostics,” Exploration (Beijing) 2 (2022): 20210171, 10.1002/EXP.20210171. 37324583 PMC10190949

[44] J. Lv , L. Chen , Y. Zhu , L. Hou , and Y. Liu , “Promoting Epithelium Regeneration for Esophageal Tissue Engineering through Basement Membrane Reconstitution,” ACS Applied Materials & Interfaces 6 (2014): 4954–4964, 10.1021/am4059809. 24679268

[45] G. Liu , X. Huang , Q. Pu , et al., “Re‐Characterization of Hammerhead Ribozymes as Molecular Tools for Intermolecular RNA Cleavage,” Organic & Biomolecular Chemistry 15 (2017): 4681–4685, 10.1039/C7OB00995J. 28517012

[46] M. Cagel , E. Grotz , E. Bernabeu , A. M. Moretton , and A. D. Chiappetta , “Doxorubicin: Nanotechnological Overviews From Bench to Bedside,” Drug Discovery Today 22 (2017): 270–281, 10.1016/j.drudis.2016.11.005. 27890669

[47] S. P. Binks and M. Dobrota , “Kinetics and Mechanism of Uptake of Platinum‐Based Pharmaceuticals by the Rat Small Intestine,” Biochemical Pharmacology 40 (1990): 1329–1336, 10.1016/0006-2952(90)90400-F. 2206139

[48] R. Suginaka , R. Izui , J. Inoue , et al., “Induction of Apoptosis in Human Pancreatic Carcinoma Cells by a Synthetic Bleomycin‐Like Ligand,” Japanese Journal of Cancer Research 89 (1998): 947–953, 10.1111/j.1349-7006.1998.tb00653.x. 9818031 PMC5921946

[49] J. Portugal , “Challenging Transcription by DNA‐Binding Antitumor Drugs,” Biochemical Pharmacology 155 (2018): 336–345, 10.1016/j.bcp.2018.07.030. 30040927

[50] R. K. Mishra and L. Maganti , “Antitumor Drugs Effect on the Stability of Double‐Stranded DNA: Steered Molecular Dynamics Analysis,” Journal of Biomolecular Structure & Dynamics 40 (2022): 11373–11382, 10.1080/07391102.2021.1960193. 34355668

[51] J. Shang , Y. Qiao , G. Mao , L. Qian , G. Liu , and H. Wang , “Bleomycin‐Fe(II) Agent with Potentiality for Treating Drug‐Resistant H1N1 Influenza Virus: A Study Using Electrochemical RNA Beacons,” Analytica Chimica Acta 1180 (2021): 338862, 10.1016/j.aca.2021.338862. 34538316

[52] K. Wong , Y. Liu , M. Wong , and J. Liu , “Cornea‐SELEX for Aptamers Targeting the Surface of Eyes and Liposomal Drug Delivery,” Exploration (Beijing) 4 (2024): 20230008, 10.1002/EXP.20230008. 39175889 PMC11335462

[53] Z. Nourian , W. Roelofsen , and C. Danelon , “Triggered Gene Expression in Fed‐Vesicle Microreactors With a Multifunctional Membrane,” Angewandte Chemie International Edition 51 (2012): 3114–3118, 10.1002/anie.201107123. 22246637

[54] K. Nishimura , S. Tsuru , H. Suzuki , and T. Yomo , “Stochasticity in Gene Expression in a Cell‐Sized Compartment,” ACS Synthetic Biology 4 (2015): 566–576, 10.1021/sb500249g. 25280237

[55] M. L. Jacobs , M. A. Boyd , and N. P. Kamat , “Diblock Copolymers Enhance Folding of a Mechanosensitive Membrane Protein During Cell‐Free Expression,” PNAS 116 (2019): 4031–4036, 10.1073/pnas.1814775116. 30760590 PMC6410776

[56] H. Jespersen , J. H. Andersen , H. J. Ditzel , and O. G. Mouritsen , “Lipids, Curvature Stress, and the Action of Lipid Prodrugs: Free Fatty Acids and Lysolipid Enhancement of Drug Transport Across Liposomal Membranes,” Biochimie 94 (2012): 2–10, 10.1016/j.biochi.2011.07.029. 21839138

[57] D. Garenne and V. Noireaux , “Analysis of Cytoplasmic and Membrane Molecular Crowding in Genetically Programmed Synthetic Cells,” Biomacromol 21 (2020): 2808–2817, 10.1021/acs.biomac.0c00513. 32441931

[58] K. P. Adamala , D. A. Martin‐Alarcon , K. R. Guthrie‐Honea , and E. S. Boyden , “Engineering Genetic Circuit Interactions Within and Between Synthetic Minimal Cells,” Nature Chemistry 9 (2017): 431–439, 10.1038/nchem.2644. PMC5407321 28430194

[59] C. Hu , R. Fang , B. Luk , and L. Zhang , “Polymeric Nanotherapeutics: Clinical Development and Advances in Stealth Functionalization Strategies,” Nanoscale 6 (2014): 65–75, 10.1039/C3NR05444F. 24280870

[60] J. Wang , X. Hu , C. Han , S. Hou , H. Wang , and F. Zheng , “Lanthanide Complexes for Tumor Diagnosis and Therapy by Targeting Sialic Acid,” ACS Nano 16 (2022): 14827–14837, 10.1021/acsnano.2c05715. 35981089

[61] J. Chen , X. Yu , X. Liu , J. Ni , G. Yang , and K. Zhang , “Advances in Nanobiotechnology‐Propelled Multidrug Resistance Circumvention of Cancer,” Nanoscale 14 (2022): 12984–12998, 10.1039/D2NR04418H. 36056710

[62] C. Wang , F. Li , T. Zhang , M. Yu , and Y. Sun , “Recent Advances in Anti‐Multidrug Resistance for Nano‐Drug Delivery System,” Drug Delivery 29 (2022): 1684–1697, 10.1080/10717544.2022.2079771. 35616278 PMC9154776

[63] P. S. Steeg , “Targeting Metastasis,” Nature Reviews Cancer 16 (2016): 201–218, 10.1038/nrc.2016.25. 27009393 PMC7055530

[64] G. Housman , S. Byler , S. Heerboth , et al., “Drug Resistance in Cancer: An Overview,” Cancers (Basel) 6 (2014): 1769–1792, 10.3390/cancers6031769. 25198391 PMC4190567

[65] N. Lyu , B. Pedersen , E. Shklovskaya , H. Rizos , M. P. Molloy , and Y. Wang , “SERS Characterization of Colorectal Cancer Cell Surface Markers Upon Anti‐EGFR Treatment,” Exploration (Beijing) 2 (2022): 20210176, 10.1002/EXP.20210176. 37323700 PMC10190927

[66] S. Kuermanbayi , Y. Yang , Y. Zhao , et al., “In Situ Monitoring of Functional Activity of Extracellular Matrix Stiffness‐Dependent Multidrug Resistance Protein 1 Using Scanning Electrochemical Microscopy,” Chemical Science 13 (2022): 10349–10360, 10.1039/D2SC02708A. 36277620 PMC9473519

[67] T. T. T. Ngo , B. Rossbach , I. Sébastien , J. C. Neubauer , A. Kurtz , and K. Hariharan , “Functional Differentiation and Scalable Production of Renal Proximal Tubular Epithelial Cells From Human Pluripotent Stem Cells in a Dynamic Culture System,” Cell Proliferation 55 (2022): e13190, 10.1111/cpr.13190. 35102634 PMC8891564

[68] J. He , X. Wang , K. Chen , M. Zhang , and J. Wang , “The Amino Acid Transporter SLC7A11‐Mediated Crosstalk Implicated in Cancer Therapy and the Tumor Microenvironment,” Biochemical Pharmacology 205 (2022): 115241, 10.1016/j.bcp.2022.115241. 36084707

[69] W. Fang , L. Li , Z. Lin , et al., “Engineered IL‐15/IL‐15R α ‐Expressing Cellular Vesicles Promote T Cell Anti‐Tumor Immunity,” Extracell Vesicle 2 (2023): 100021, 10.1016/j.vesic.2022.100021.

[70] Y. Zheng , Y. Han , Q. Sun , and Z. Li , “Harnessing Anti‐Tumor and Tumor‐Tropism Functions of Macrophages via Nanotechnology for Tumor Immunotherapy,” Exploration (Beijing) 2 (2022): 20210166, 10.1002/EXP.20210166. 37323705 PMC10190945

[71] R. S. Yadava , R. Kumar , and P. K. Yadava , “Expression of lexA Targeted Ribozyme in Escherichia coli BL‐21 (DE3) Cells,” Molecular and Cellular Biochemistry 271 (2005): 197–203, 10.1007/s11010-005-6340-6. 15881671

[72] H. Zhang , X. Zhang , X. Wu , et al., “Interference of Frizzled 1 (FZD1) Reverses Multidrug Resistance in Breast Cancer Cells through the Wnt/β‐catenin Pathway,” Cancer Letters 323 (2012): 106–113, 10.1016/j.canlet.2012.03.039. 22484497

[73] Z. Sun , J. Ge , B. Guo , et al., “Extremely Low Frequency Electromagnetic Fields Facilitate Vesicle Endocytosis by Increasing Presynaptic Calcium Channel Expression at a Central Synapse,” Scientific Reports 6 (2016): 21774, 10.1038/srep21774. 26887777 PMC4757866

[74] C. Mao , X. Liu , Y. Zhang , et al., “DHODH‐Mediated Ferroptosis Defence Is a Targetable Vulnerability in Cancer,” Nature 593 (2021): 586–590, 10.1038/s41586-021-03539-7. 33981038 PMC8895686

[75] X. Tang , T. Chen , Y. Ma , et al., “Enzyme Reaction‐Assisted Programmable Transcriptional Switches for Bioactive Molecule Detection,” Analytical Chemistry 96 (2024): 331–338, 10.1021/acs.analchem.3c04198. 38127443

