# Supplementary material for: Tumor‐Mimic Artificial Cell Integrated With In Situ Synthetic Biology for Testing of Antitumor Drug Sensitivity
Source: Exploration (Beijing). 2026 May 28;6(3):20240134. doi: 10.1002/EXP.20240134 (PMC13317558; doi:10.1002/EXP.20240134)
Supplement: Supplementary file 1 — Supporting File: exp270177‐sup‐0001‐SuppMat.pdf. [file EXP2-6-20240134-s001.pdf]

Supporting Information

Tumor-mimic artificial cell integrated with *in situ* synthetic biology for testing of antitumor drug sensitivity

Chaoyang Guan[#], Runchi Zhang[#], Zhihui Zhou, Pei Sun, Yichun Mao, Changqing Mao, Yonggeng Ma, Guifang Chen, Qiuhong Man,* and Chang Feng**

C. Guan, Z. Zhou, P. Sun, Y. Mao, C. Mao, Y. Ma, G. Chen, C. Feng
Center for Molecular Recognition and Biosensing, Shanghai Engineering Research Center of Organ Repair, Joint International Research Laboratory of Biomaterials and Biotechnology in Organ Repair (Ministry of Education), Shanghai Key Laboratory of Bio-Energy Crops, School of Life Sciences, Shanghai University, Shanghai 200444, P. R. China.
E-mail: gfchen@shu.edu.cn; cfeng@shu.edu.cn

R. Zhang
Department of Clinical Laboratory Medicine, Shanghai Tenth People's Hospital, School of Medicine, Tongji University, Shanghai 200072, P. R. China

Q. Man
Department of Clinical Laboratory Medicine, Shanghai Fourth People's Hospital, School of Medicine, Tongji University, Shanghai 200434, P. R. China
E-mail: Manqiuhong307@163.com
[#]C. Guan and R. Zhang contributed equally to this work.

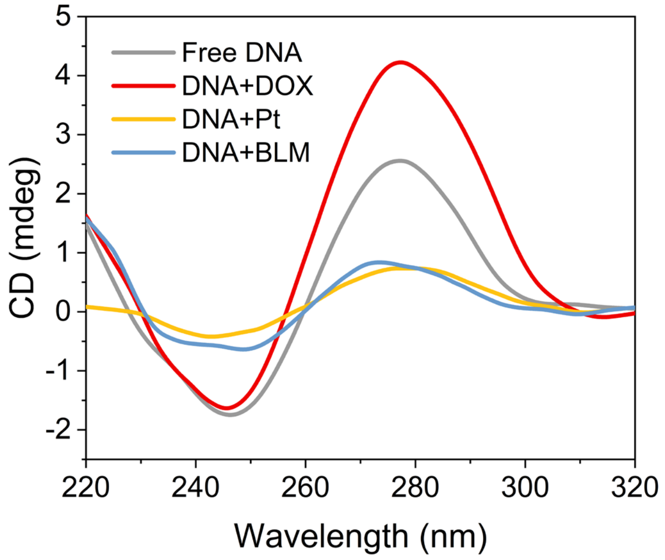

Figure S1. CD spectra of 1 μM template double-stranded DNA under the treatment of DOX (10 μM), DDP (100 μM) and BLM (100 μM).

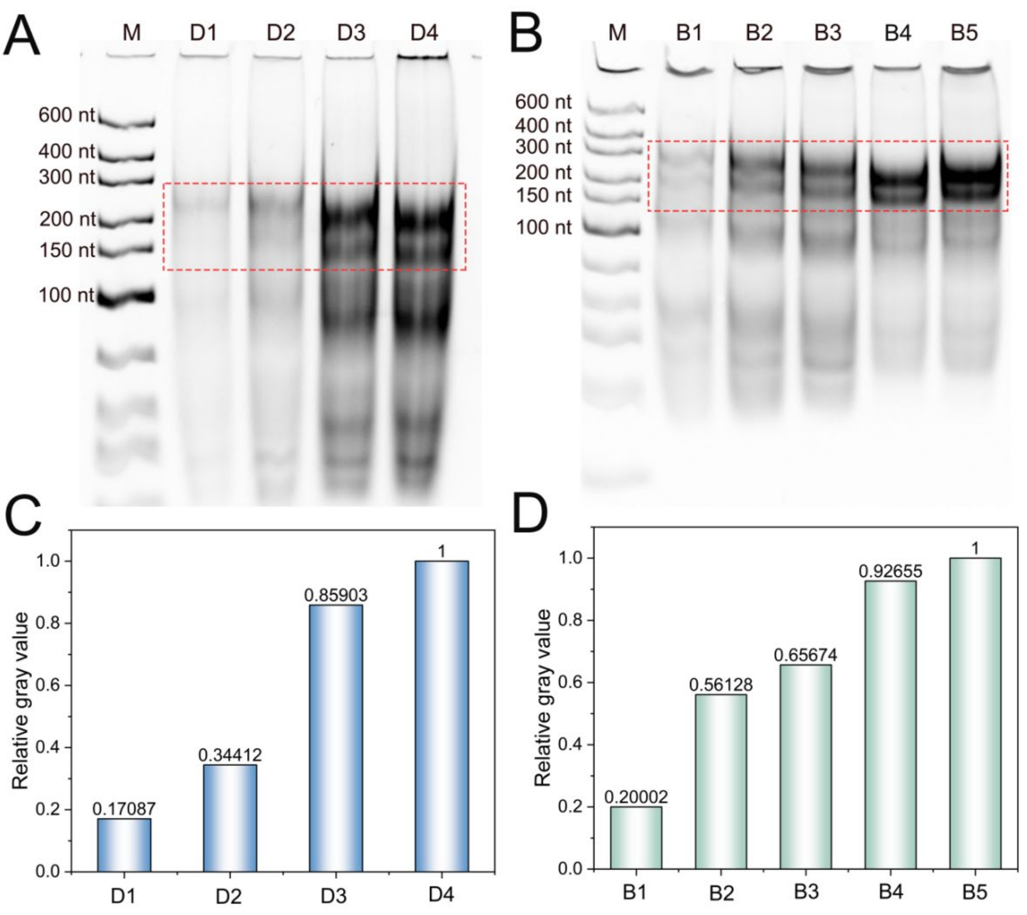

Figure S2. Electrophoretic characterization of the effects of different concentration of antineoplastic drugs on nucleic acids. (A-B) Polyacrylamide gel electrophoresis analysis of *in vitro* transcription mRNA products after antitumor drugs action. M, Marker; D1, 20 μM DOX;

D2, 10 μM DOX; D3, 2 μM DOX; D4, 0 μM DOX; B1, 200 μM BLM; B2, 100 μM BLM; B3, 20 μM BLM; B4, Fe^{2+}; B5, 0 μM BLM. (C-D) Gray value analysis of wireframe (red) in A and B, respectively. The gray value was analyzed by Image J software.

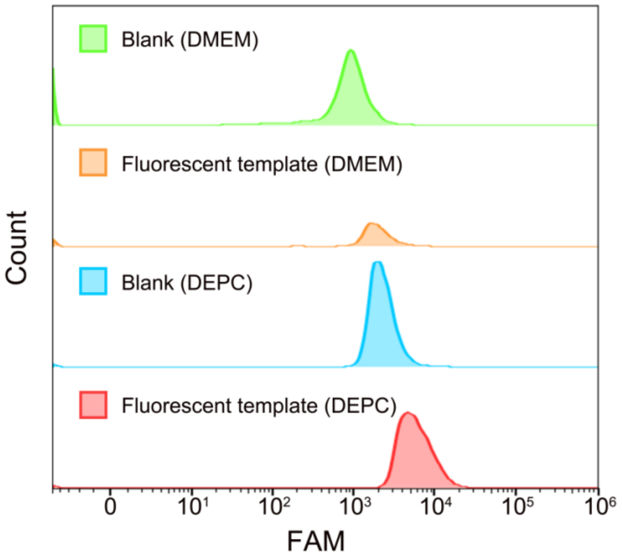

Figure S3. Fluorescence measurement for performance of LAC template DNA@LACs and template DNA-FQ@LACs in various buffer solution by using flow cytometry.

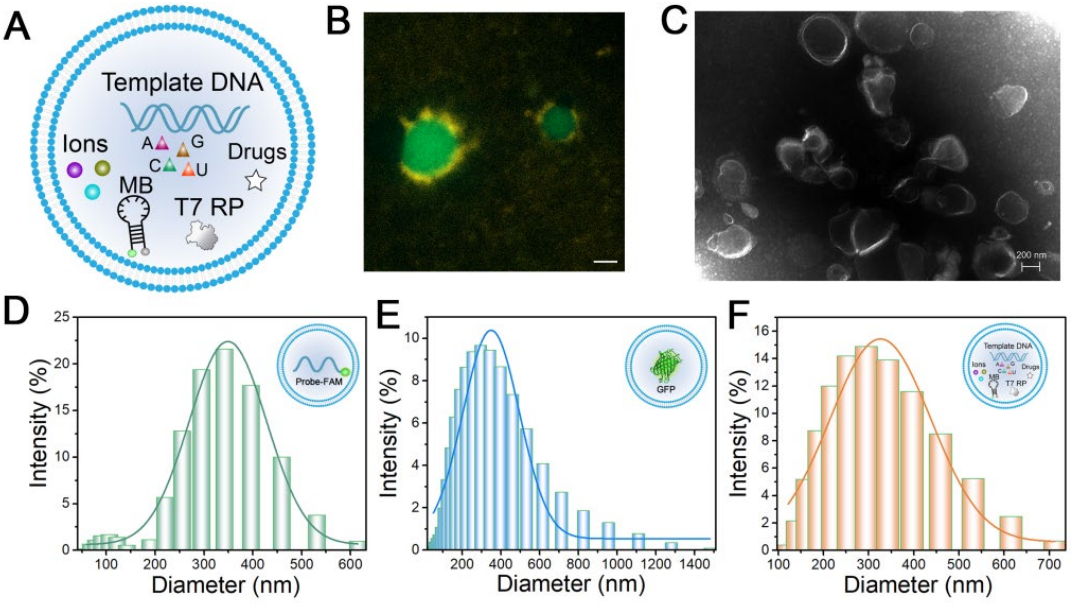

Figure S4. Characterization of artificial cells based on liposomes. (A) Schematic diagram of LAC construction based on simple phospholipid bilayer. (B) Fluorescent microscopy images of OPRS@LACs. Scale bars, 200 nm. (C) Transmission electron microscopy images showing

OPRS@LACs. Scale bars, 200 nm. (D-F) Dynamic light scattering analysis of the template DNA-FQ@LACs (D), GFP@LACs (E), OPRS@LACs (F).

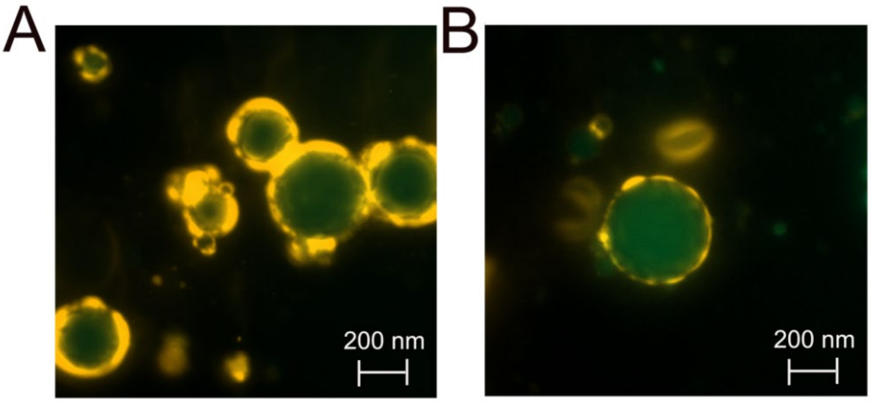

Figure S5. Fluorescent microscopy images of (A) DNA-FQ@LACs and (B) GFP@LACs. Scale bars, 200 nm.

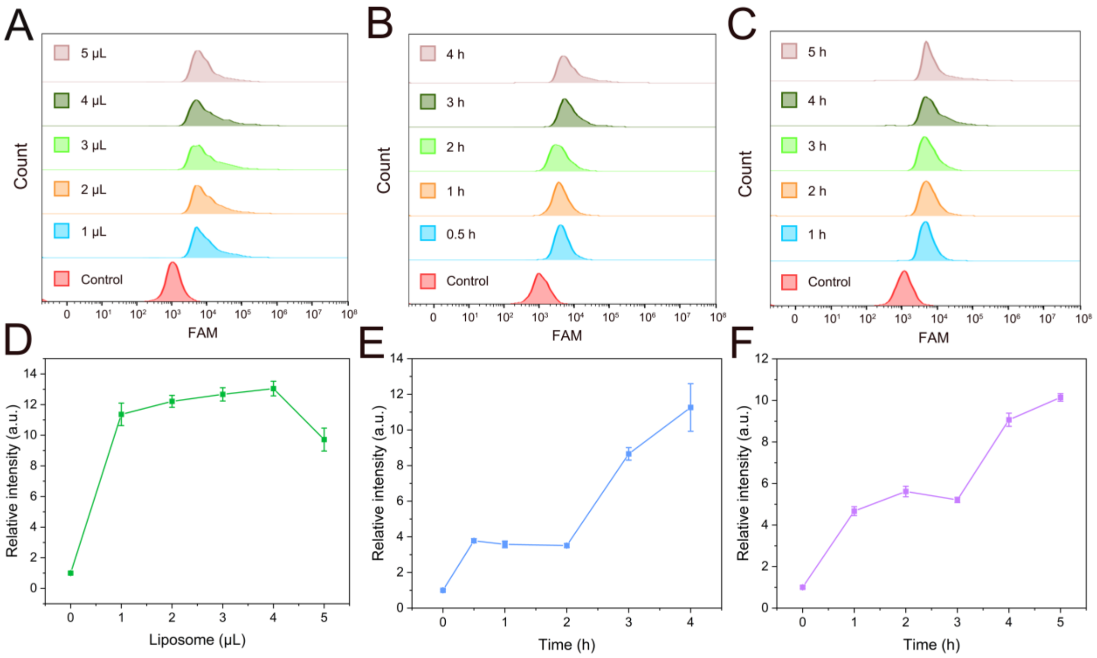

Figure S6. Optimization of LAC model. (A, D) Optimization of lipo8000 transfection reagent dosage for constructing the membrane of OPRS@LACs. (B, E) Optimization of lipo8000 transfection reagent encapsulation time for constructing OPRS@LACs. (C, F) Optimization of the time of one-pot reaction in OPRS@LACs. In D-F, all the points represent the means based on three independent replicates; bars represent the s.d.

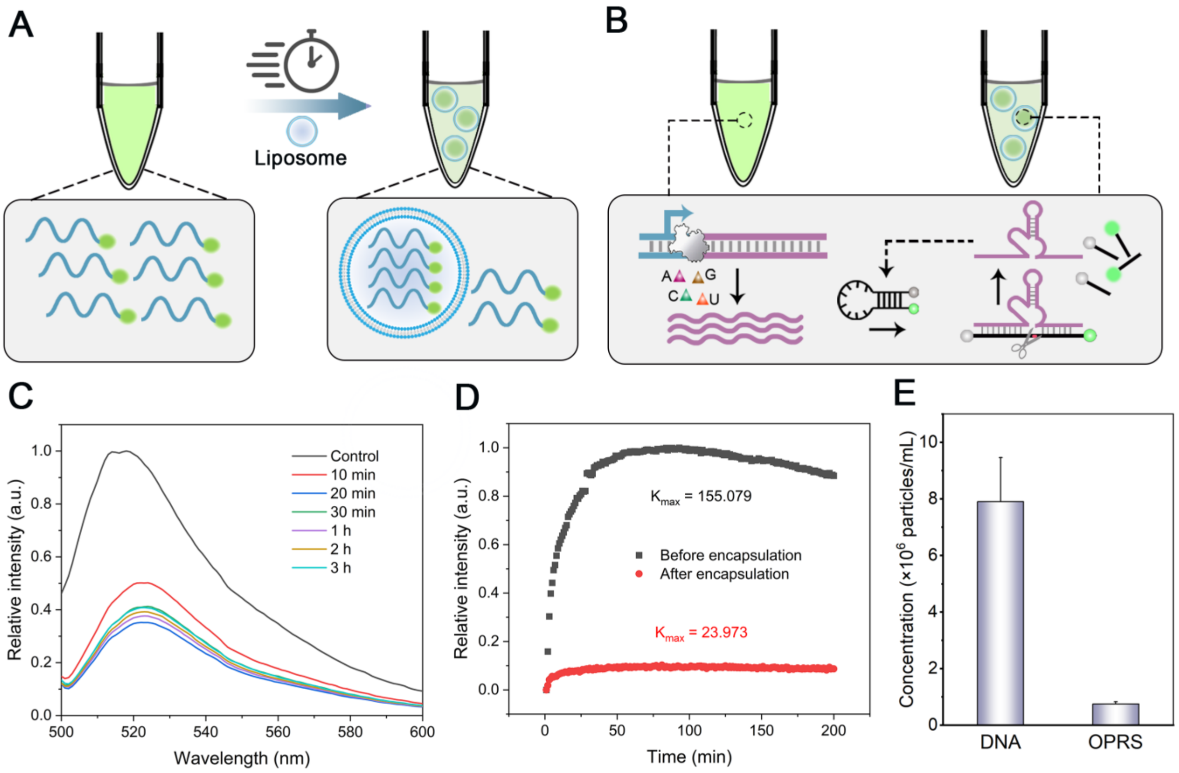

Figure S7. Calculation of encapsulation efficiency. (A-B) Schematic diagrams of incubation process for detecting encapsulation efficiency. (C) Fluorescence spectra of template DNA-FQ encapsulated by liposomes at different time. Template DNA-FQ without liposomes was set as control group. (D) Time courses of fluorescence synthesized by one-pot reaction in solution and OPRS@LACs. (E) NTA characterizes the density of template DNA-FQ@LACs and OPRS@LACs. In E, each data point represents the average of two independent experiments ± SD.

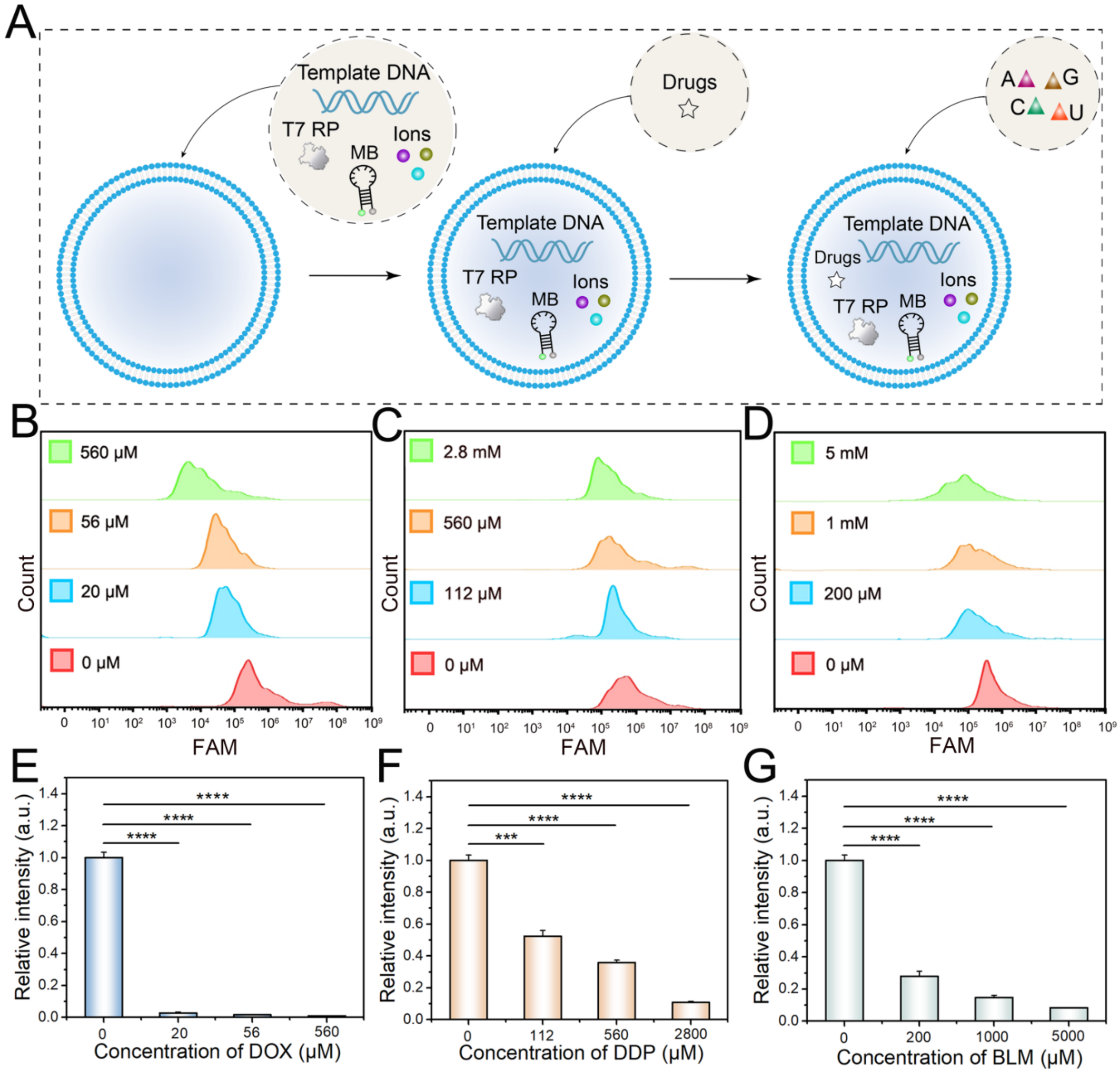

Figure S8. Testing the sensitivity of antitumor drugs by LAC model. (A) Schematic illustration of antitumor drug sensitivity test performed by OPRS@LACs. (B-D) Representative flow cytometric profiles in OPRS@LACs at different concentration of DOX, DDP and BLM. (E-G) Normalized fluorescence intensity at different concentration of DOX, DDP and BLM. The fluorescence value was analyzed by FlowJo v10 software. In E-G, each data point represents the average of three independent experiments ± SD. Statistical significance was determined by two-tailed Student's t-test; ***$p < 0.001$, ****$p < 0.0001$.

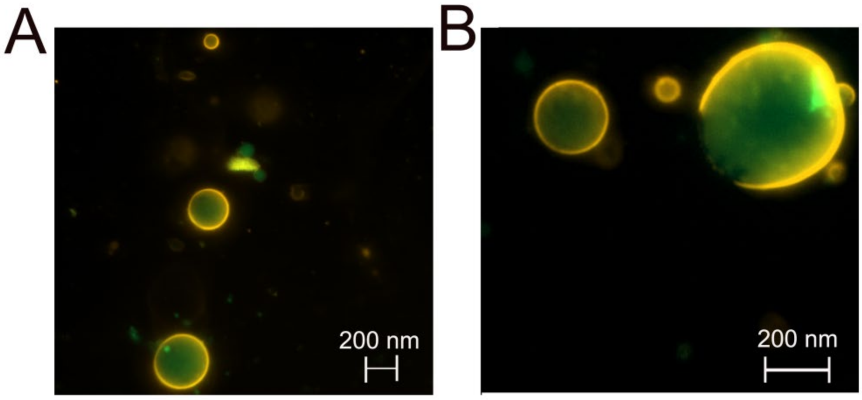

Figure S9. Fluorescent microscopy images of (A) DNA-FQ@CCMACs and (B) GFP@CCMACs. Scale bars, 200 nm.

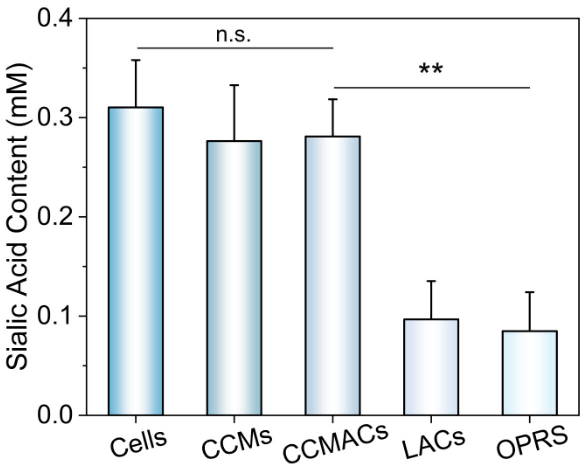

Figure S10. Determination of sialic acid content on membrane surface. All the points represent the means based on three independent replicates; bars represent the s.d. Statistical significance was determined by two-tailed Student's t-test; $**p < 0.01$; n.s., not significant ($p > 0.05$).

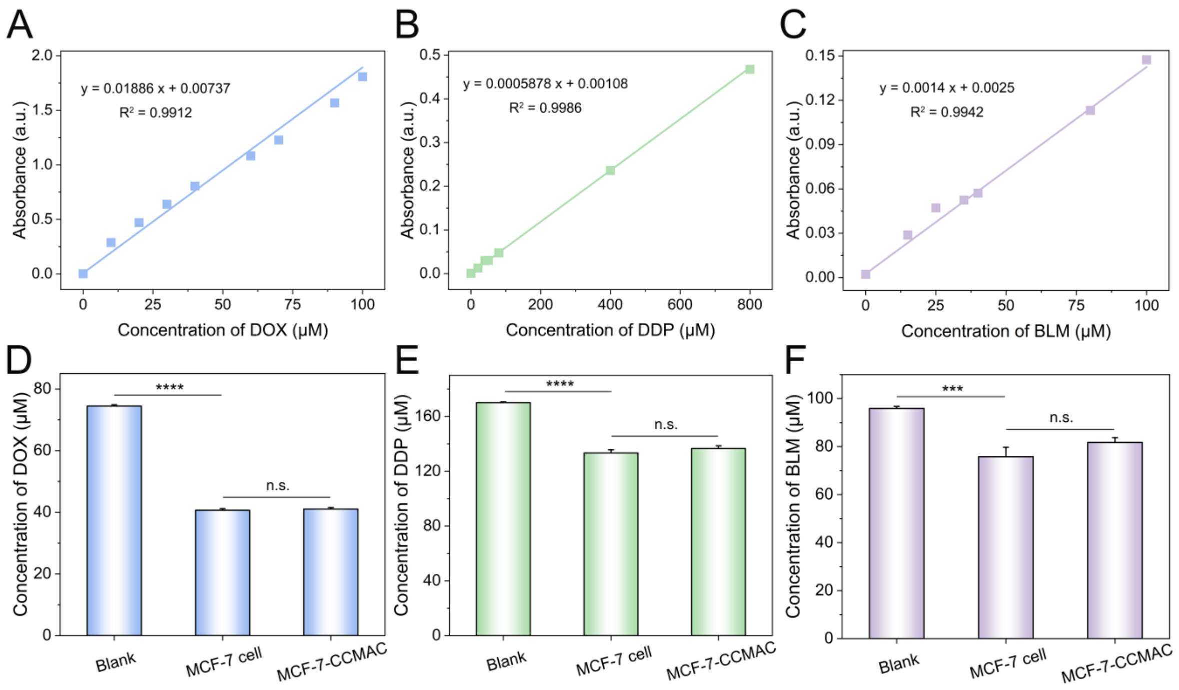

Figure S11. Comparison of drug uptake between CCMACs and tumor cells. (A-C) Standard curves of characteristic peak for concentration of DOX, DDP and BLM. (D-F) Comparison of the residual concentrations of DOX, DDP and BLM after uptake by CCMACs and tumor cells with the blank group. All the points represent the means based on three independent replicates; bars represent the s.d. Statistical significance was determined by two-tailed Student's t-test; ***$p < 0.001$, ****$p < 0.0001$; n.s., not significant ($p > 0.05$).

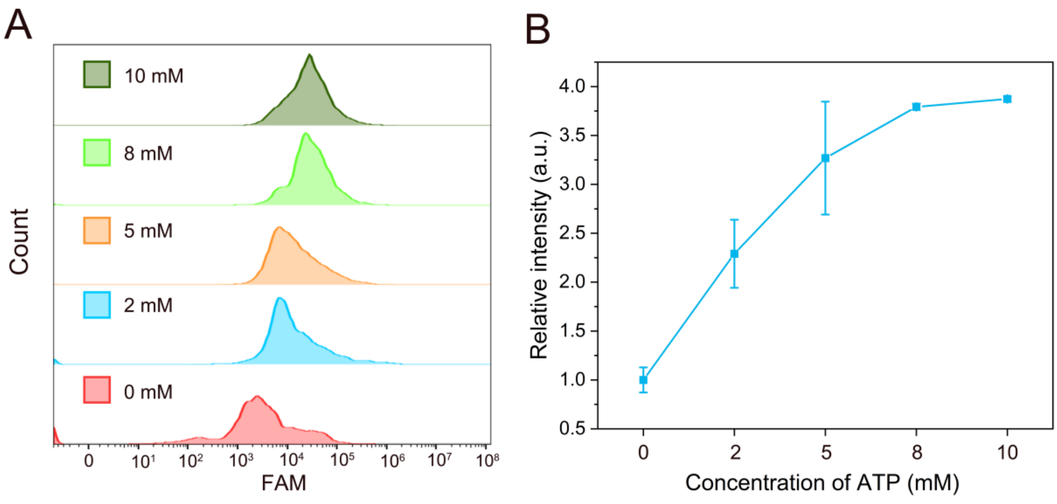

Figure S12. Optimization of the concentration of ATP as an auxiliary small molecule during the sensitivity test of antitumor drugs *via* CCMACs. In B, all the points represent the means

based on two independent replicates; bars represent the s.d.

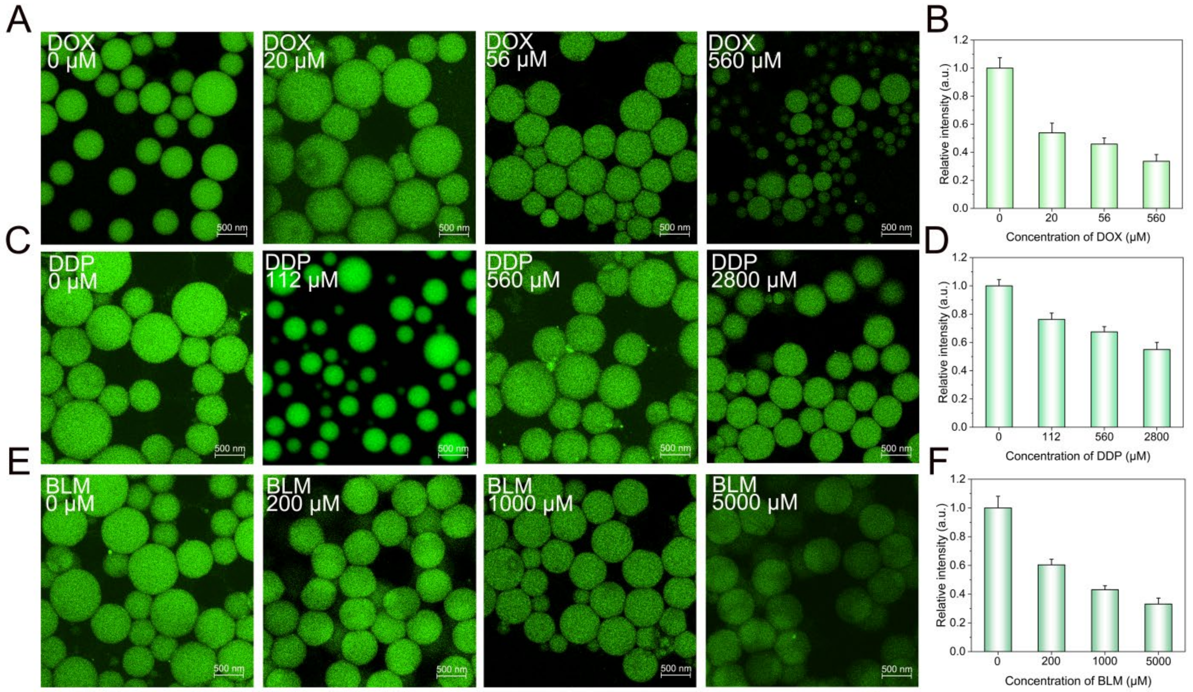

Figure S13. Confocal images showing the sensitivity of DOX (A,B), DDP (C,D) and BLM (E,F) in HeLa-CCMAC. n = 5. Scale bars, 500 nm.

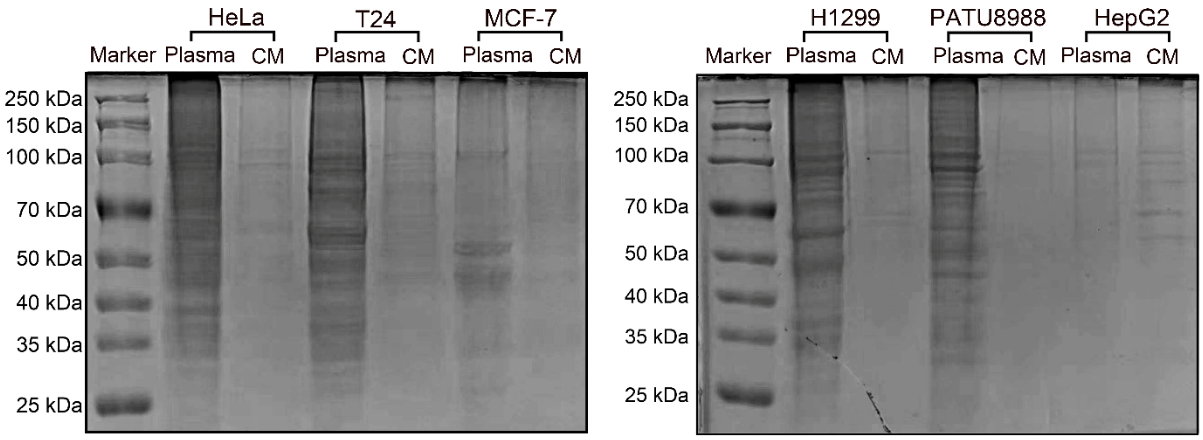

Figure S14. Coomassie blue staining was used to characterize the isolated cell membrane proteins and cytoplasmic proteins of HeLa, T24, MCF-7, H1299, PATU8988, HepG2.

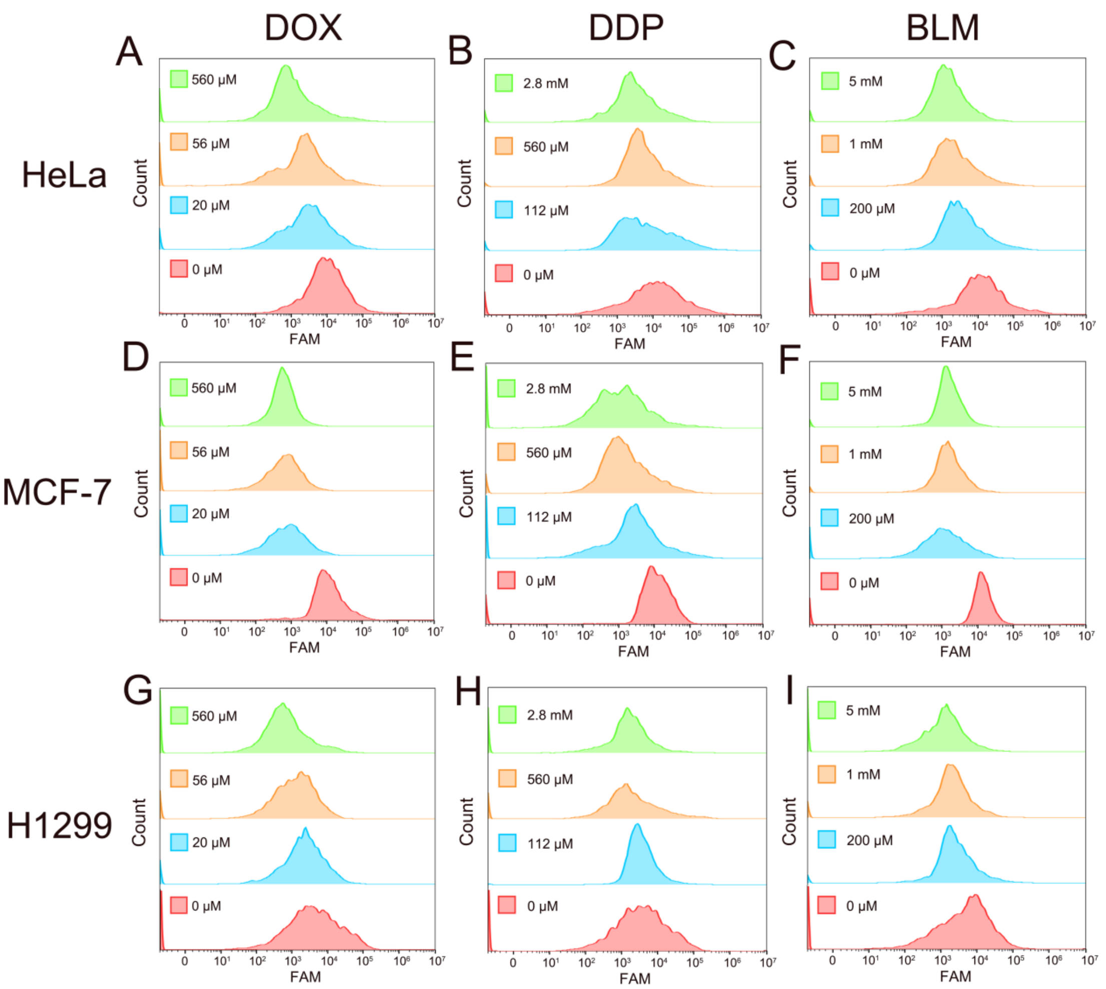

Figure S15. Representative flow cytometric profiles at different concentration of DOX (A, D, G), DDP (B, E, H), and BLM (C, F, I) in HeLa-CCMAC, MCF-7-CCMAC, H1299-CCMAC.

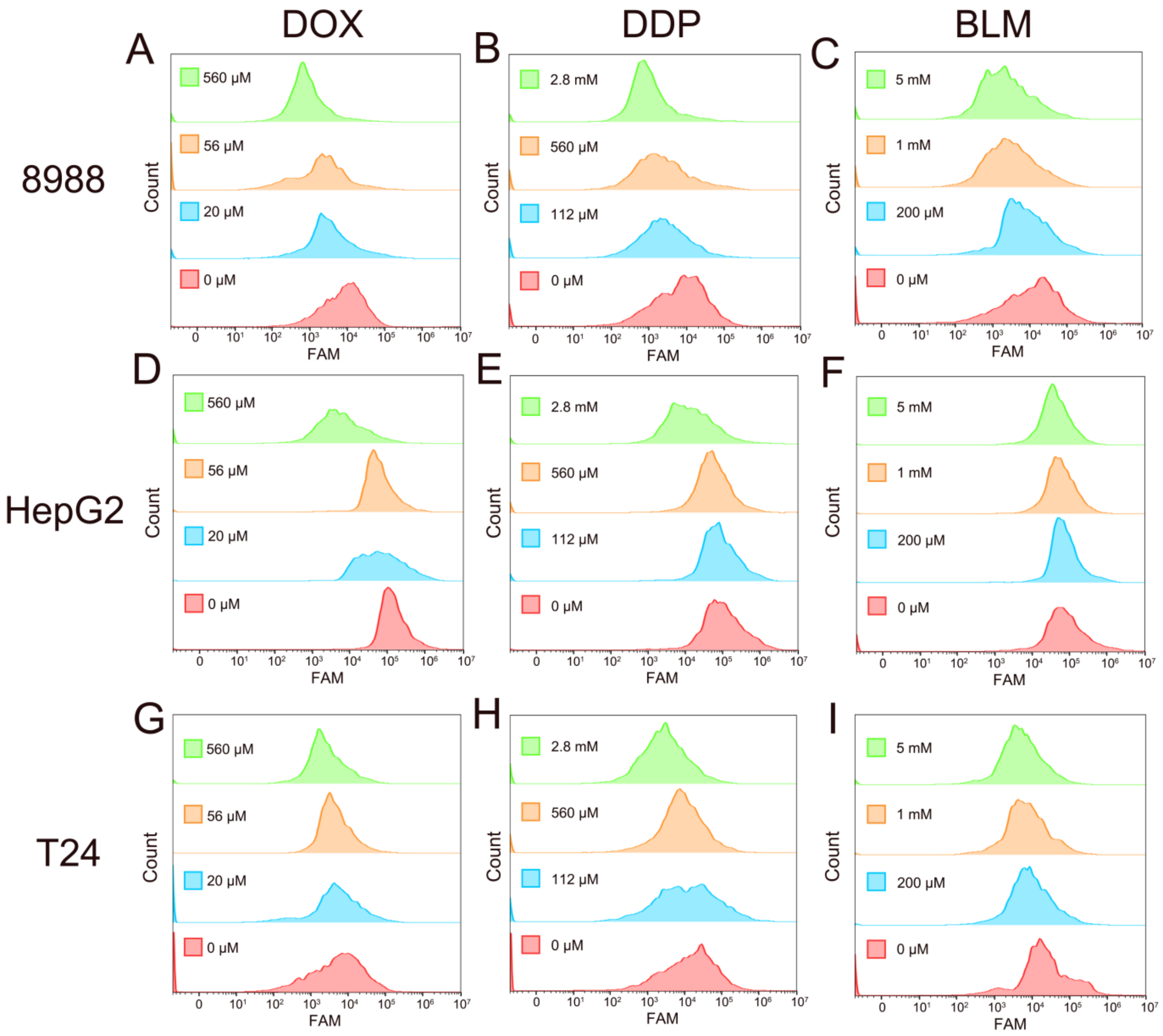

Figure S16. Representative flow cytometric profiles at different concentration of DOX (A, D, G), DDP (B, E, H), and BLM (C, F, I) in PATU8988-CCMAC, HepG2-CCMAC, T24-CCMAC.

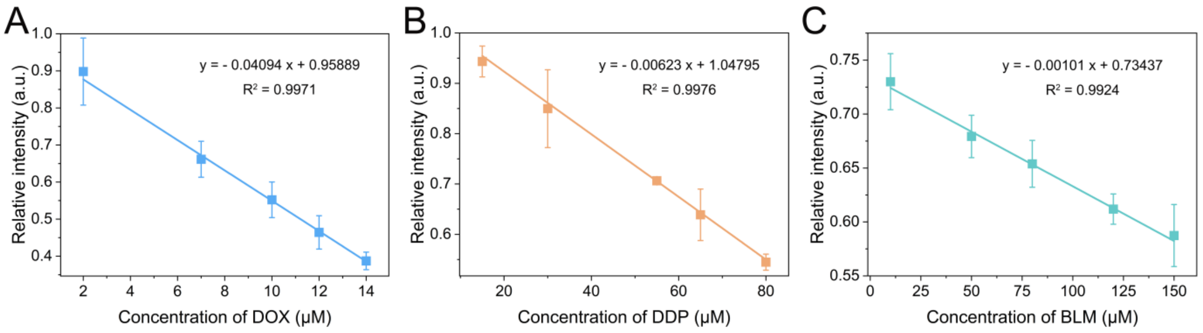

Figure S17. Sensitivity test of MCF-7-CCMAC to DOX (A), DDP (B) and BLM (C) in low concentration range. All the points represent the means based on three independent replicates;

bars represent the s.d.

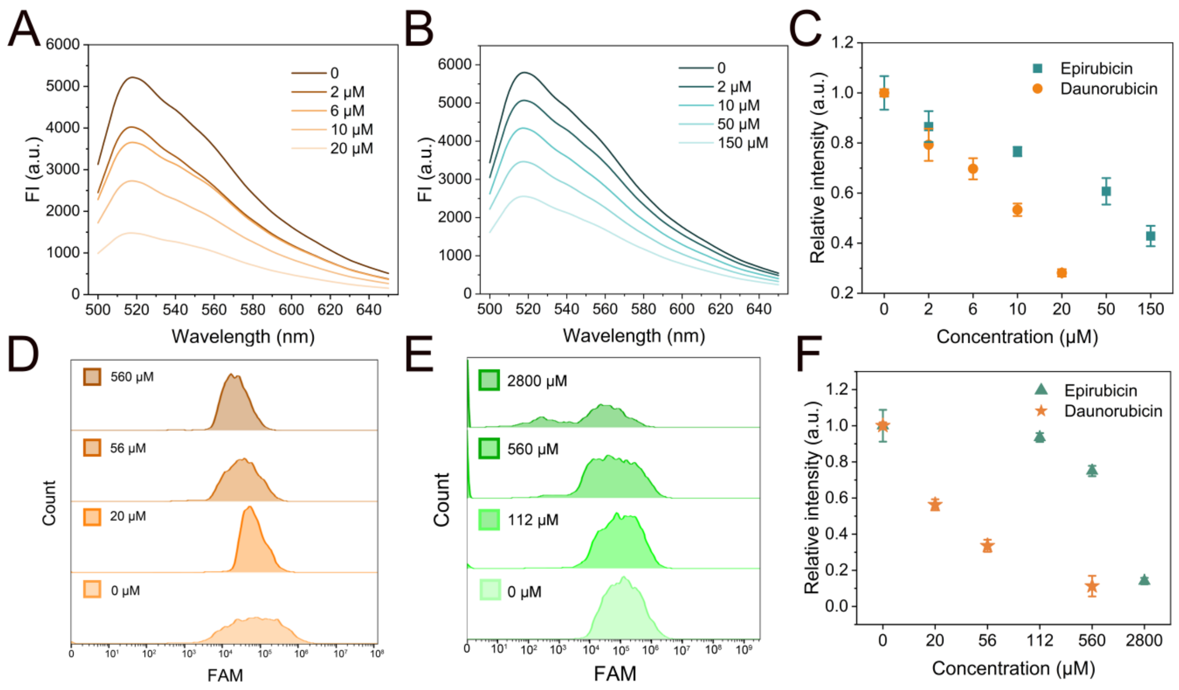

Figure S18. Efficacy test of epirubicin and daunorubicin in solution and HeLa-CCMACs. (A-B) Fluorescence spectra of different concentration of epirubicin and daunorubicin. (C) The difference of corresponding normalized fluorescence intensity at different concentration of epirubicin and daunorubicin by one-pot reaction in solution. (D-E) Representative flow cytometric profiles in OPRS@CCMACs at different concentration of epirubicin and daunorubicin. (F) Normalized fluorescence results of HeLa-CCMAC for antitumor drugs sensitivity evaluation. The fluorescence value was analyzed by FlowJo v10 software. In C and F, all the points represent the means based on three independent replicates; bars represent the s.d.

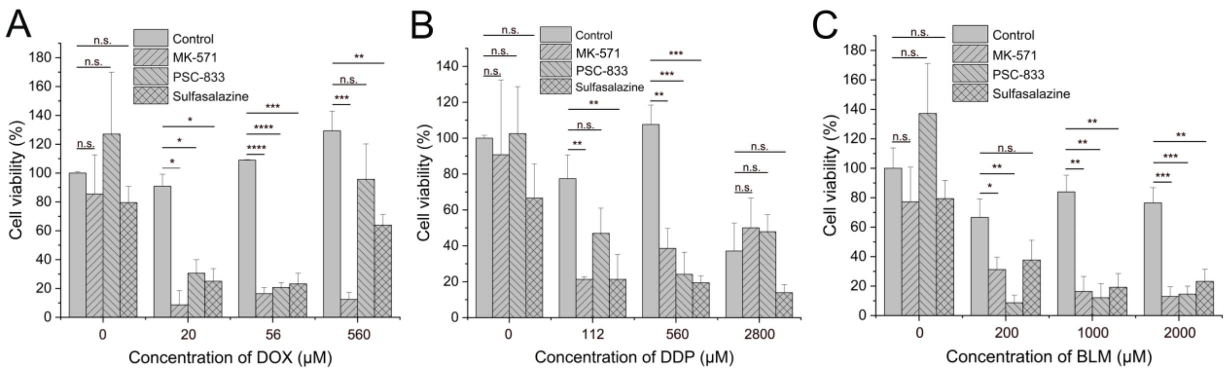

Figure S19. Cell toxicity analysis. T24 cells were treated with MK-571, PSC-833, sulfasalazine. Then treated with different concentration of DOX (A), DDP (B), BLM (C). Cell viability was assessed by CCK8 assay. All the points represent the means based on three independent replicates; bars represent the s.d. Statistical significance was determined by two-tailed Student's t-test; *p < 0.05, **p < 0.01, ***p < 0.001, ****p < 0.0001; n.s., not significant (p > 0.05).

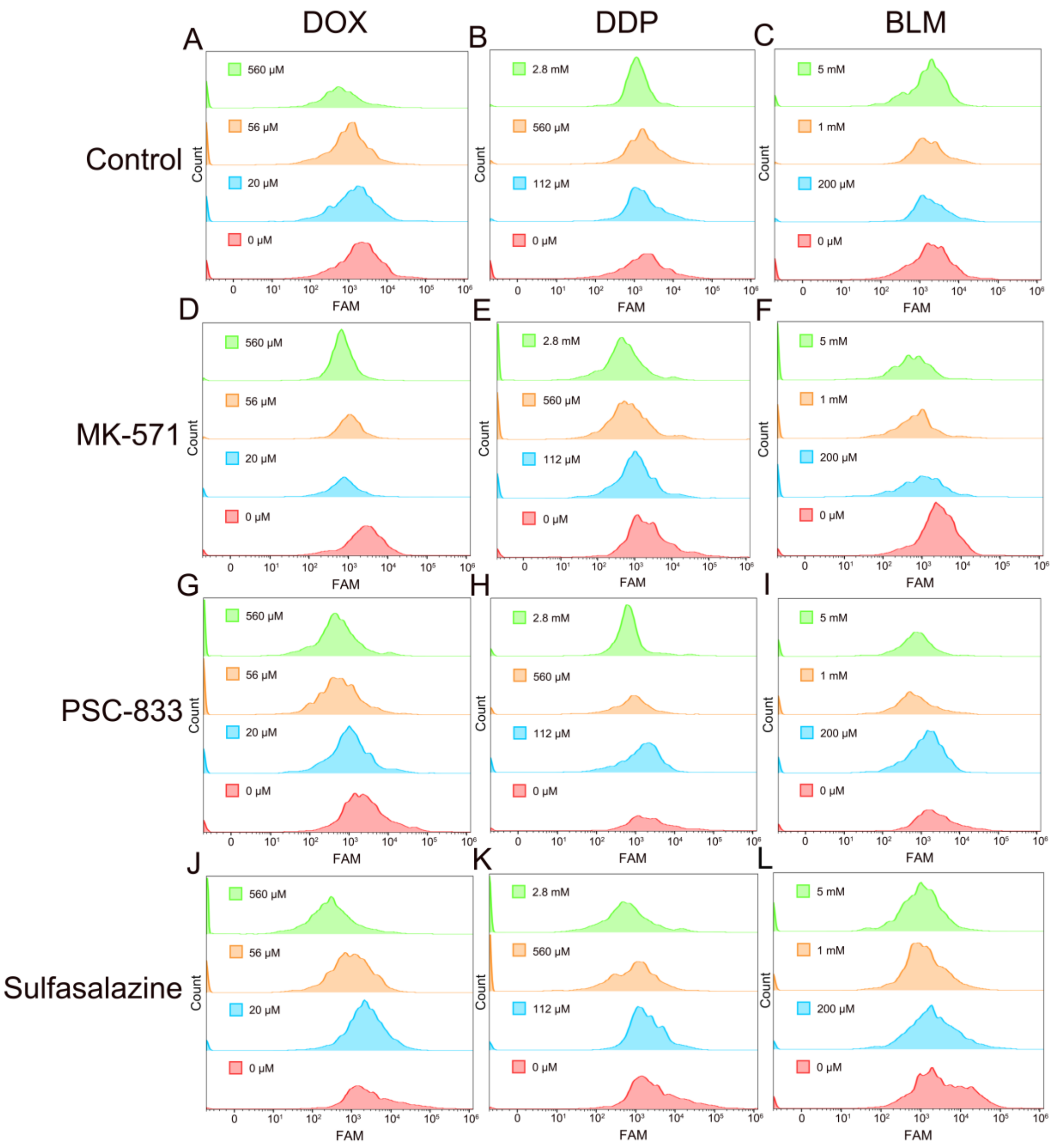

Figure S20. Representative flow cytometric profiles at different concentration of DOX (A, D, G, J), DDP (B, E, H, K), and BLM (C, F, I, L) in T24-CCMAC treated with MK-571, PSC-833, sulfasalazine.

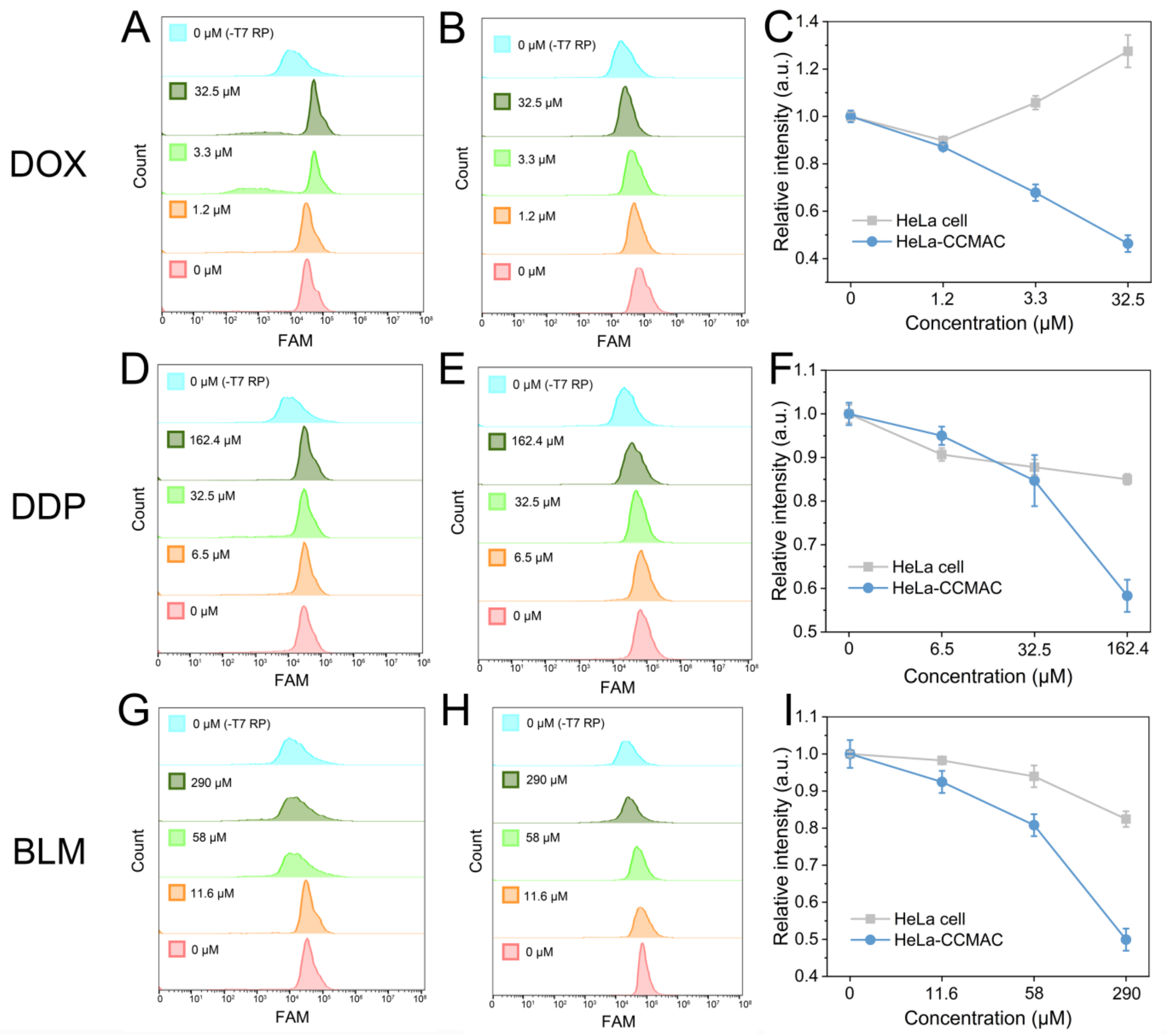

Figure S21. Representative flow cytometric profiles in HeLa cell and HeLa-CCMACs at different concentration of DOX (A-B), DDP (D-E), and BLM (G-H). (C, F, I) Comparison of normalized fluorescence results of HeLa cell and HeLa-CCMACs for antitumor drugs sensitivity evaluation. The fluorescence value was analyzed by FlowJo v10 software. In C, F and I, all the points represent the means based on three independent replicates; bars represent the s.d.

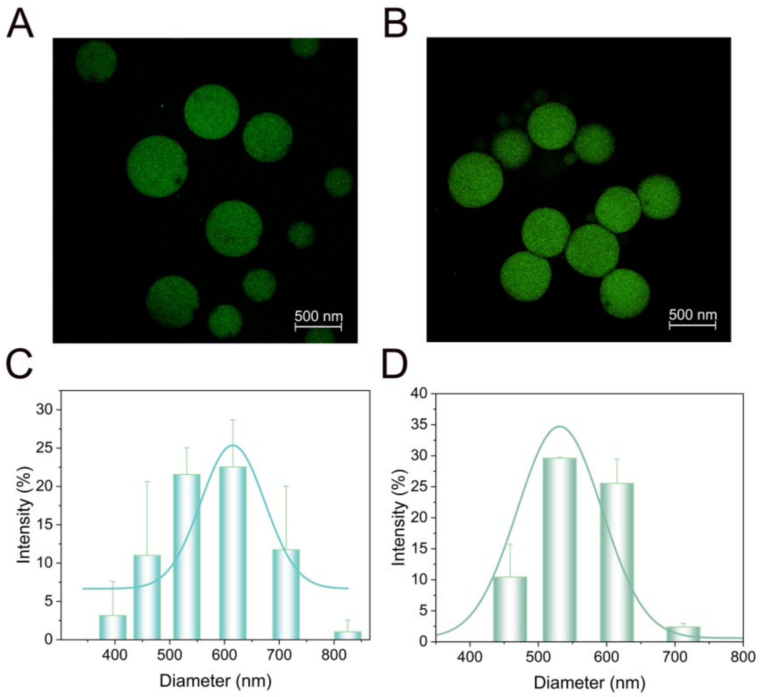

Figure S22. Characterization of OPRS@MDA-MB-231-CCMAC and OPRS@MIA-PACA-2-CCMAC membranes derived from PDX tissue cells. (A, B) Confocal images of OPRS@MDA-MB-231-CCMAC (A) and OPRS@MIA-PACA-2-CCMAC (B). Scale bars, 500 nm. (C, D) Dynamic light scattering analysis of OPRS@MDA-MB-231-CCMAC (C) and OPRS@MIA-PACA-2-CCMAC (D). In C and D, all the points represent the means based on two independent replicates; bars represent the s.d.

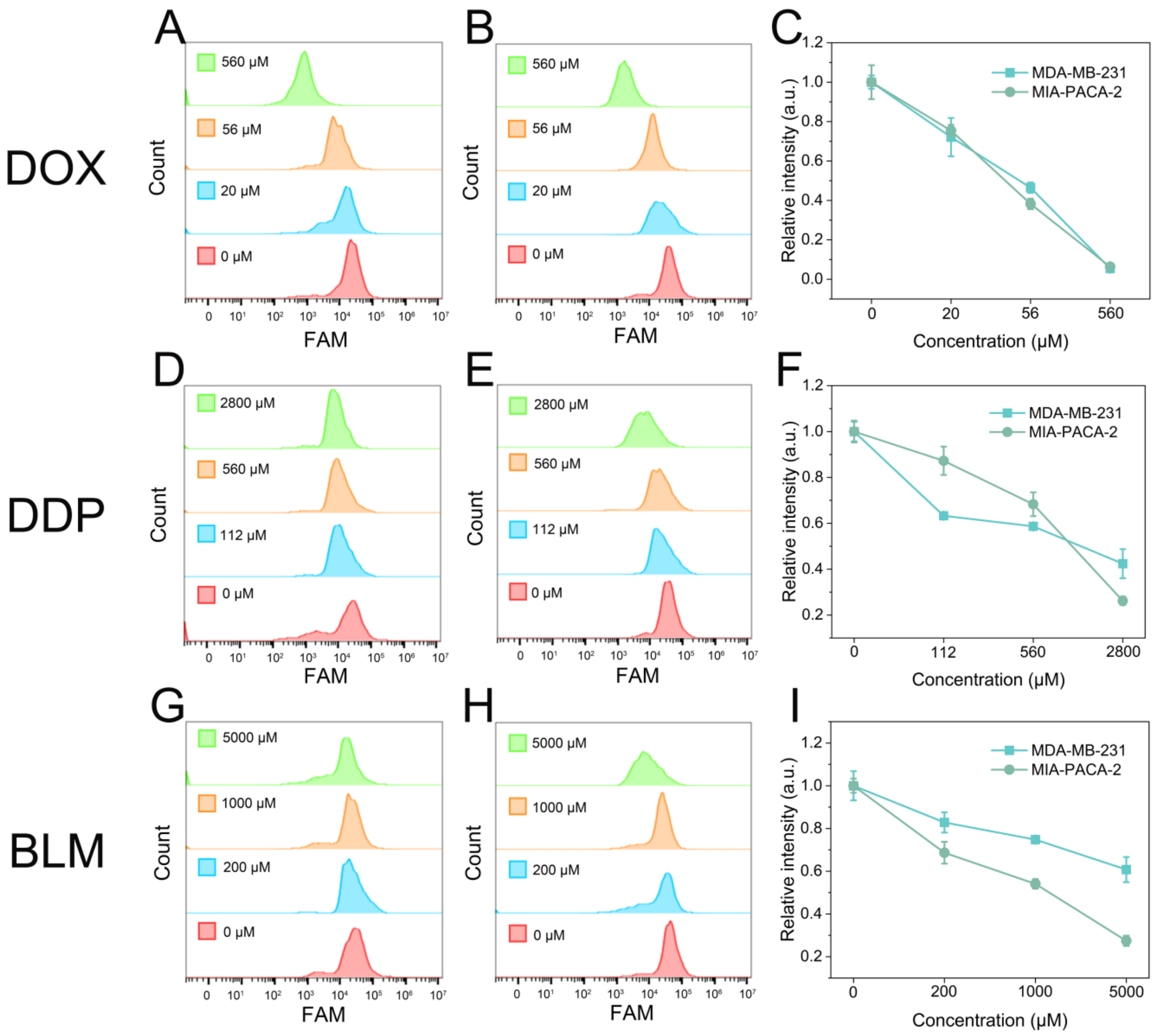

Figure S23. Comparison of normalized fluorescence results of MDA-MB-231-CCMAC and MIA-PACA-2-CCMAC for DOX (A-C), DDP (D-F) and BLM (G-I) sensitivity evaluation. The fluorescence value was analyzed by FlowJo v10 software. In C, F and I, all the points represent the means based on three independent replicates; bars represent the s.d.

Table S1. Oligonucleotides used in this study.

Oligonucleotide	Sequence (5'→3')	Modification
Template DNA (sense strand)	TAA TAC GAC TCA CTA TAG GGG AAT TGT GAG CGG ATA ACA ATT CCG GCA GCC ACA CCT GAT GAG TCC GTG AGG ACG AAA CCT ACC AG	
Template DNA (antisense strand)	CTG GTA GGT TTC GTC CTC ACG GAC TCA TCA GGT GTG GCT GCC GGA ATT GTT ATC CGC TCA CAA TTC CCC TAT AGT GAG TCG TAT TA	
Molecular probe (MB)	AAAAA CUGGUAGGUC GUGUGGCUGCC AAAAA	5'-FAM 3'-BHQ
FAM-Template DNA (sense strand)	TAA TAC GAC TCA CTA TAG GGG AAT TGT GAG CGG ATA ACA ATT CCG GCA GCC ACA CCT GAT GAG TCC GTG AGG ACG AAA CCT ACC AG	5'-FAM
